# Nanoparticle-based optical interfaces for retinal neuromodulation: a review

**DOI:** 10.3389/fncel.2024.1360870

**Published:** 2024-03-20

**Authors:** Paul R. Stoddart, James M. Begeng, Wei Tong, Michael R. Ibbotson, Tatiana Kameneva

**Affiliations:** ^1^School of Science, Computing and Engineering Technologies, Swinburne University of Technology, Hawthorn, VIC, Australia; ^2^Department of Biomedical Engineering, Faculty of Engineering & Information Technology, The University of Melbourne, Melbourne, VIC, Australia; ^3^School of Physics, The University of Melbourne, Melbourne, VIC, Australia

**Keywords:** neuromodulation, retinal prosthesis, nanoparticle transducers, optical nanosensors, retinal pharmacokinetics, retinal degeneration

## Abstract

Degeneration of photoreceptors in the retina is a leading cause of blindness, but commonly leaves the retinal ganglion cells (RGCs) and/or bipolar cells extant. Consequently, these cells are an attractive target for the invasive electrical implants colloquially known as “bionic eyes.” However, after more than two decades of concerted effort, interfaces based on conventional electrical stimulation approaches have delivered limited efficacy, primarily due to the current spread in retinal tissue, which precludes high-acuity vision. The ideal prosthetic solution would be less invasive, provide single-cell resolution and an ability to differentiate between different cell types. Nanoparticle-mediated approaches can address some of these requirements, with particular attention being directed at light-sensitive nanoparticles that can be accessed via the intrinsic optics of the eye. Here we survey the available known nanoparticle-based optical transduction mechanisms that can be exploited for neuromodulation. We review the rapid progress in the field, together with outstanding challenges that must be addressed to translate these techniques to clinical practice. In particular, successful translation will likely require efficient delivery of nanoparticles to stable and precisely defined locations in the retinal tissues. Therefore, we also emphasize the current literature relating to the pharmacokinetics of nanoparticles in the eye. While considerable challenges remain to be overcome, progress to date shows great potential for nanoparticle-based interfaces to revolutionize the field of visual prostheses.

## 1 Introduction

Sight is a major source of sensory input for humans, and the sense that people most fear losing. Some of the most common causes of blindness are degenerative diseases of the outer retina, like age-related macular degeneration (AMD) and retinitis pigmentosa (RP). AMD is the leading cause of blindness in people aged 50 years and older in high-income countries (Chaudhuri et al., 2023), while RP is the leading cause of visual disability and blindness in those younger than 60 years old, affecting over 1.5 million people worldwide (Verbakel et al., 2018). Conventional retinal prostheses generally aim to bypass the photoreceptors and restore vision in blind patients by electrically stimulating the surviving inner retinal neurons with an electrode array. In particular, the retinal ganglion cells (RGCs) and their optic nerve projections largely maintain their axonal connections to the lateral geniculate nucleus and other visual centers in the brain, thus preserving their ability to convey visual information despite the loss of the photoreceptors. An artificial sense of vision can be provided by translating visual scenes into appropriate spatio-temporal patterns of retinal neuronal activity. This has been an active area of research since 1967 (Brindley and Lewin, 1968).

**Figure 1:**
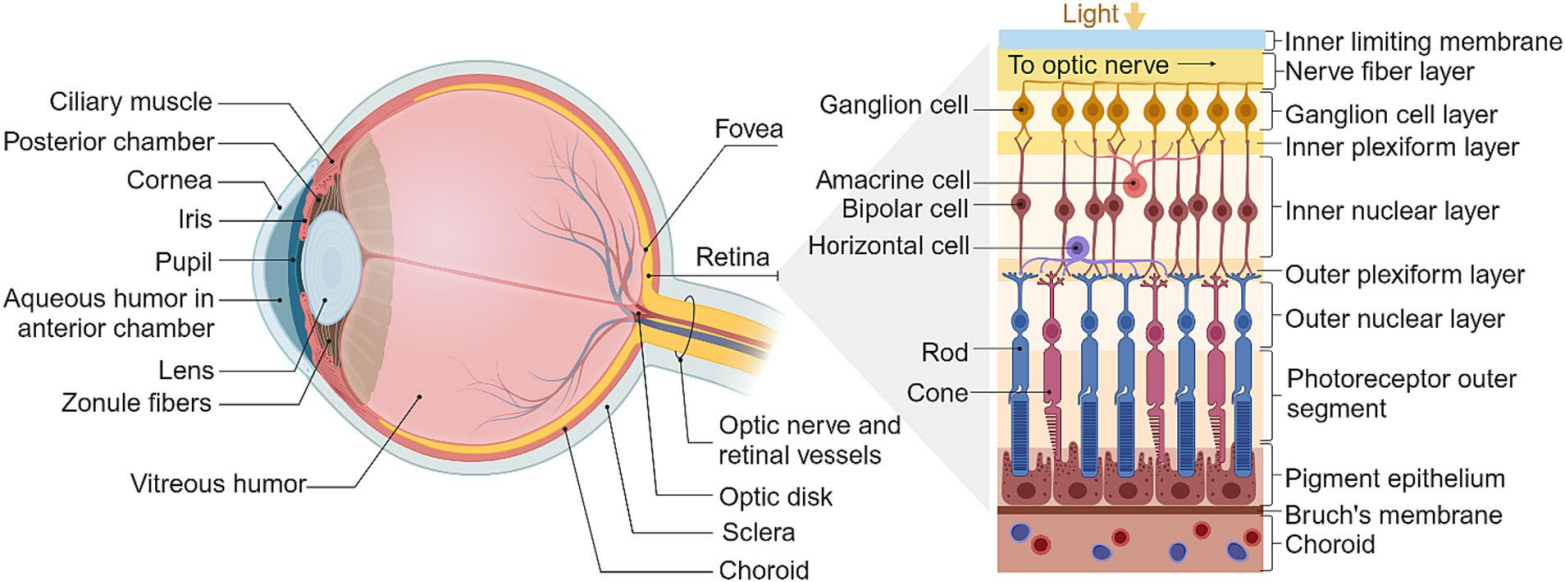
The structure of the eye and retina. Adapted from Kandel (2014), created in BioRender.com (2023).

Patients implanted with retinal prostheses report the appearance of bright, blurred spots in their visual fields in response to electrical stimulation. These bright regions are termed phosphenes, and loosely appear in areas of higher stimulation current on the electrode array (Ayton et al., 2014; Kameneva et al., 2016). Thus, modern stimulation strategies attempt to construct greyscale images from areas of high and low injected current, implementing increasingly high electrode densities in order to construct finer image structures. Neuroprosthetic visual implants can be inserted epiretinally (on the inner surface of the retina), subretinally (between retina and retinal pigment epithelium, RPE) or suprachoroidally (between sclera and choroid) as outlined in Figure 1. In practice, higher electrode densities have not translated effectively into finer spatial resolution (Ayton et al., 2014; Maturana et al., 2016). This is primarily due to the issue of current spread, where the isotropic dispersion of electrical current into proximal tissue has a tendency to stimulate large populations of RGCs. This current spread phenomenon can be reduced by lowering the injected current amplitude but must still exceed the RGC stimulation threshold. Novel stimulation strategies such as current shaping (Spencer et al., 2019; Tong et al., 2020a) and materials such as diamond electrodes (Tong et al., 2020b) may provide some improvement in percept resolution in future devices but have not yet been implemented clinically.

In bypassing the low-level retinal processing which would be present in a healthy retina, retinal prostheses can also lose a significant amount of visual information, such as color and contrast sensitivity. In particular, if the ON–OFF bipolar network is bypassed, electrical prostheses stimulate both ON and OFF RGCs indiscriminately; since these pathways encode the opposite sensations of brightness and darkness respectively, stimulation of both ON and OFF cells at the same time manifests as a significant reduction in image contrast (Ayton et al., 2014; Maturana et al., 2016). To date, only people with degenerative retinal diseases have been eligible to receive a retinal prosthesis. The technology is also expensive, costing patients around $150,000 in the United States (Strickland and Harris, 2022). These various limitations have had a negative effect on patient acceptance, with both Second Sight Medical Products (manufacturer of the Argus II), and Retina Implant AG (manufacturer of the Alpha IMS) ceasing to produce and implant their devices in 2019 (Ayton et al., 2020a; Strickland and Harris, 2022).

Despite these setbacks, the intrinsic optical access of the extant eye makes it an excellent candidate for less invasive optical modulation technologies. Investigation into the optical manipulation of neurons has been ongoing for several decades, and spans the ultraviolet (Booth et al., 1950), visible (Fork, 1971) and infrared (Wells et al., 2005) spectral ranges. In modern prosthesis design, optical neuromodulation offers a solution to at least two key drawbacks of electrical stimulation. Firstly, removal or distancing of the interface allows optical technologies to obviate the issue of reduced stimulation sensitivity due to scarring (Richter et al., 2011). Secondly, with the exception of certain opto-electric technologies (Wang and Guo, 2016; Wang et al., 2022), optical stimulation techniques do not exhibit current spread (Dieter et al., 2019) and thus offer a theoretically diffraction-limited stimulus resolution. However, for wavelengths that are strongly absorbed in tissue, the optical neuromodulation interface may also have to be implanted, in which case many of the same translational challenges arise as for electrical interfaces, including biocompatibility, thermal loading, portability, reliability and manufacturing scale-up (Hart et al., 2019). Consequently researchers have also considered ultrasonic (Norton, 2003; Naor et al., 2016; Darrow, 2019) and magnetic fields (Bonmassar et al., 2012) as less invasive alternatives.

A variety of nanoparticle interfaces have been reported that can transduce optical, acoustic and magnetic fields into neural electrical responses. In essence, the nanoparticles act as highly localized sensors for the externally applied field and efficiently transduce the energy to achieve a neuromodulatory response. In the context of retinal neuromodulation, optically-active nanoparticles have generated rapidly growing interest as a means to potentially bypass the natural photoreceptors while localizing the light interactions and reducing off-target effects. This review will emphasize these recently emerging approaches, placed within the broader context of other less invasive optical, ultrasonic and magnetic techniques for retinal modulation. To highlight areas where further research may advance the field, we have included some examples of nanoparticle-based tools that show promise for retinal neuromodulation but may not yet have been applied in the retina.

While the topic of retinal stimulation strategies to restore vision has been thoroughly reviewed (Yue et al., 2016; Bar-Noam and Shoham, 2020; Farnum and Pelled, 2020; Nowik et al., 2020; Shim et al., 2020; Ayton et al., 2020a) and there are several broad-based reviews for optical neuromodulation (Richter et al., 2011; Thompson et al., 2014; Richardson et al., 2020; Park et al., 2021; Jiang et al., 2022; Karatum et al., 2023) and nanoparticle-mediated neuromodulation (Wang and Guo, 2016; Paviolo and Stoddart, 2017; Wang Y. C. et al., 2018; Ledesma et al., 2019; Li et al., 2021; Benfenati and Lanzani, 2021a; Shi et al., 2022; Yang et al., 2023), none of these have discussed optically-mediated, nanoparticle-based techniques in the specific context of the retina. A recent review by Liu et al. (2022) was primarily concerned with the application of nanotechnology in implantable interfaces, whereas the focus here is on nanoparticles as a minimally-invasive optical interface. Since the retina is naturally adapted for optical access, the emphasis here is on optically-responsive nanoparticles. Other less invasive neuromodulation techniques, such as optogenetics, infrared neural modulation and ultrasonic neuromodulation, are discussed here to the extent that they provide important context for the optically-addressed nanoparticle equivalents. By the same token, those nanotransducers that are directly addressed by magnetic (Huang et al., 2010; Farnum and Pelled, 2020; Romero et al., 2022) or ultrasonic fields (Cafarelli et al., 2021) are only considered briefly here for completeness.

We note that optically-responsive nanoparticle-based approaches remain broadly compatible with existing head-mounted cameras, light projection goggles and signal processing capacity that have been developed in support of bioelectronic (Ayton et al., 2020b) or optogenetic approaches (Reutsky-Gefen et al., 2013; Sahel et al., 2021). The requirements for light projection and image processing to appropriately encode information to the retina for correct interpretation in the visual cortex have been comprehensively discussed (Bar-Noam and Shoham, 2020). Therefore, we instead focus here on the outstanding pharmacokinetic challenges that relate to nanoparticle delivery and cell-specific targeting in the retina. While the broader questions of nanoparticle synthesis and chemistry (Chen et al., 2016), surface functionalization (Mout et al., 2012), safety (Wolfram et al., 2015) and pharmacokinetics in the retina (del Amo et al., 2017; Loftsson, 2022; Tawfik et al., 2022) have been comprehensively reviewed, here we instead consider what is currently known about nanoparticle delivery to the retina, which may be useful for clinical translation of nanoparticle optical interfaces in the future.

## 2 The retina and its degeneration

The healthy retina is comprised of five major cell types stratified into three cellular layers, along with two synaptic layers and an additional five ancillary layers (Kandel, 2014), as summarized in Figure 1. Phototransduction occurs in the outermost cellular layer of the retina, which contains the photoreceptor rods and cones. These neurons hyperpolarize in response to light and are synaptically connected to bipolar cells which in turn transmit to RGCs in the innermost cellular layer. Horizontal cells and amacrine cells are typically co-stratified with bipolar cell bodies and afferent bipolar synapses, and shape the receptive fields of their associated ganglion cells through a diverse range of mechanisms such as lateral inhibition and color selectivity (Kolb et al., 1995; Kandel, 2014; Willermain et al., 2014).

The RGCs are the output layer of the retina. These cells transmit information to various visual centers of the brain via the optic nerve. In mammals, most of the optic nerve axons converge on an area of the thalamus called the dorsal lateral geniculate nucleus, which projects its own axons to the primary visual cortex. A minority of optic nerve axons also connect directly to specialized subcortical structures which play a role in various autonomic aspects of vision (Dowling and Dowling, 2016).

**Figure 2:**
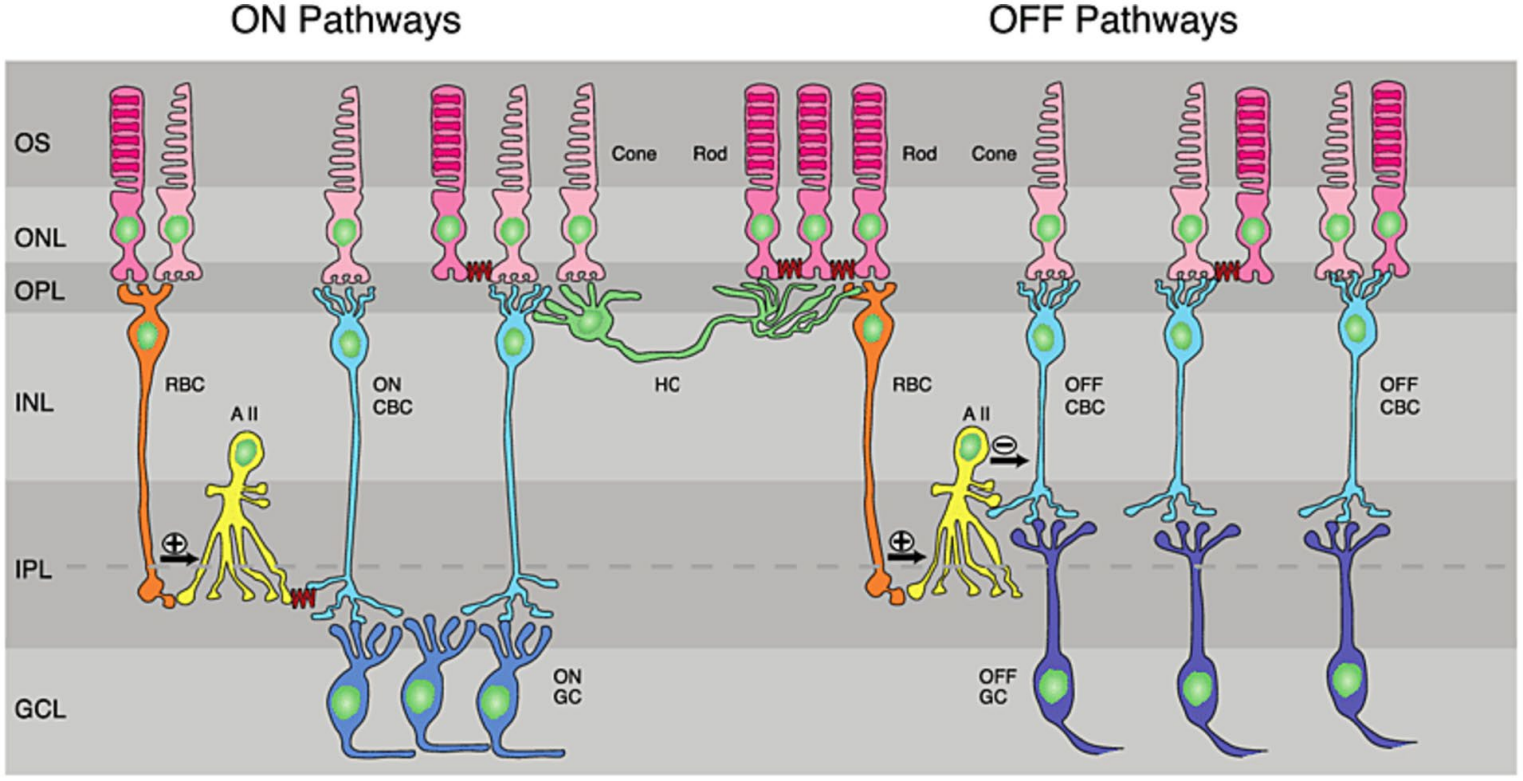
Retinal circuitry: ON and OFF pathways in the retina. RBC, rod bipolar cell; CNC, cone bipolar cell; HC, horizontal cell; AII, AII amacrine cells; GC, ganglion cell; IPL, inner plexiform layer; GCL, ganglion-cell layer; INL, inner nuclear layer; ONL, outer nuclear layer; OPL, outer plexiform layer; OS, outer segments of photoreceptor. Reproduced under the terms of the Creative Commons Attribution License, from Fain and Sampath (2018).

Different visual features are encoded by different types of neurons in the retina. Photoreceptors transmit their visual information to RGCs through a complex circuitry of bipolar, amacrine and horizontal neurons (Kandel, 2014). The exact roles that this circuitry plays in image pre-processing are incredibly diverse and an area of active research, but can broadly be described by the pathways illustrated in Figure 2. The sign of the RGC output classifies it as either an ON or OFF RGC: ON RGCs have low intrinsic activity at rest and depolarize in response to light, whilst OFF RGCs exhibit high intrinsic activity in darkness and hyperpolarize in response to light. Although ON and OFF RGCs display distinct intrinsic electrophysiology (Margolis and Detwiler, 2007; Wong et al., 2012), the ON and OFF pathways are already defined at the level of bipolar cell synapses (Kandel, 2014). A subset of ganglion cells, described as ON–OFF cells, collect both ON and OFF signals and contributed approximately 28% of surveyed RGCs in a study of Long-Evans rats (Wong et al., 2012).

While ON and OFF RGCs respond to light in opposite fashion, these neurons are stimulated simultaneously with conventional electrical stimulation. This may lead to the cancelation of the signal transmitted to the brain, loss of edge detection, and difficulties in contrast perception. Selective activation of ON and OFF pathways is an area of active research in electrically-mediated protheses (Margolis and Detwiler, 2007; Freeman et al., 2010; Kameneva et al., 2011; Twyford et al., 2014; Kameneva et al., 2016; Muralidharan et al., 2020); however, none of these strategies have been implemented in practice. Nanoparticle-mediated retinal protheses offer an opportunity to selectively activate ON and OFF RGCs by targeting nanoparticles to different cell types. Some progress in this direction has been made by Begeng et al. (2023), where it has been shown that OFF RGCs can be preferentially inhibited using gold nanorods and long near-infrared pulses. The mechanism of inhibition during nanoparticle-enhanced infrared neuromodulation is due to the phenomenon known as thermal block, mediated by voltage-gated potassium channels suppressing action potentials at elevated temperatures (Ganguly et al., 2019). Thermal block is discussed in more detail in Sections 3.2 and 4.1. Directing nanoparticles to targets only expressed in OFF-type RGCs may allow for more robust OFF-type inhibition. It is worth noting, that none of the existing visual prostheses can inhibit neurons using conventional electrical stimulation strategies. A capacity for inhibition may provide an advantage of nanoparticle-based optical interfaces for retinal neuromodulation.

Together with ON and OFF cell types, there are more than 50 neuronal types in the mammalian retina, each type encoding different features of a visual scene (Masland, 2001a,b). Future work will require an exhaustive study of distinct RGC responses based on cell type and subtype in order to elucidate avenues for selective nanoparticle-based optical neuromodulation. In particular, by developing nanoparticles conjugated to particular cell-specific ligands, it may be possible to develop neuromodulation strategies for cellular targets. For example, there is evidence for the non-uniform distribution of transient receptor potential vanilloid 4 (TRPV4) in mammalian RGCs, which may allow for preferential stimulation of large-soma neurons (Gao et al., 2019). An improved understanding of the role of TRPV4 channels during optical neuromodulation is likely to be a necessary next step in optimizing laser stimulation protocols. In addition to selective neuromodulation, functionalized nanoparticles may offer longer stability *in vivo* as they can withstand washout (Carvalho-de-Souza et al., 2015), and lower stimulation threshold since they can be designed to form a close association with the cell membrane (Carvalho-de-Souza et al., 2019).

Due to the anatomy of the retina, there is unavoidable stimulation of passing axons in the nerve fiber layer, when an electrical implant is positioned epiretinally. This results in elongated percepts and low-resolution blurred vision. Axonal stimulation has been shown to be a significant confounding factor to existing electrical stimulation strategies (Tong et al., 2020a), and nanoparticle targeting of a membrane protein not expressed in axons, may reduce or eliminate axonal excitation during retinal stimulation. Some known strategies for targeting nanoparticles to RGCs *in vivo* are discussed in Sections 4 and 6.

During the retinal degeneration of AMD and RP, a substantial retinal remodeling occurs, starting with small-scale changes in the inner retina and atrophy of rods and cones, and then progressing to re-wiring of the whole retinal circuitry and neuronal loss (Marc and Jones, 2003; Jones et al., 2016; Pfeiffer et al., 2016; Telias et al., 2020; Pfeiffer et al., 2020a). Retinal remodeling following photoreceptor loss has been shown in rodents (Cuenca et al., 2004; Specht et al., 2007) and humans (Marc and Jones, 2003), with three phases of retinal degeneration having been observed in these studies (Marc and Jones, 2003): Phase I usually refers to the loss of rods, while Phase II denotes the loss of cones. During these phases, some amacrine cells form synapses with horizontal cells and some rod bipolar cells form gap-junctions with AII amacrine cells (Pfeiffer et al., 2020b). These connections are not present in healthy retina (refer to Figure 2). Phase III presents significant reorganization throughout the retinal circuitry, neuronal migration between retina layers, formation of new synapses, and cell death.

In addition to remodeling, rhythmic neuronal activity has been recorded in the degenerate retina following photoreceptor loss (Stasheff, 2008; Margolis et al., 2014; Euler and Schubert, 2015). Two types of rhythmic spiking have been recorded in animal studies: 3 Hz oscillations in the outer retina (Haq et al., 2014), and ~ 10 Hz spiking activity in the inner retina, particularly in electrically-coupled bipolar and AII amacrine cells, as well as RGCs (Borowska et al., 2011; Margolis et al., 2014). Retinal remodeling and changes in the intrinsic spontaneous rate of retinal neurons present significant challenges for both electrical implants and nanoparticle-based interfaces. To increase the efficacy of visual prostheses in this case, selective modulation of multiple individually-addressable RGCs may offer some advantages, since this strategy does not rely on the survival of the lower-level retinal circuitry, and the neuronal signal will be transmitted to the optic nerve directly.

## 3 Less invasive neuromodulation strategies

### 3.1 Optogenetics

Amongst optical neuromodulation methods, optogenetics has received considerable attention recently (Klapper et al., 2016; Bar-Noam and Shoham, 2020; Lindner et al., 2022; Sakai et al., 2022; Parnami and Bhattacharyya, 2023; Stefanov and Flannery, 2023). Apart from photoreceptors, most mammalian neurons are not inherently sensitive to light. Optogenetics refers to the process of genetically modifying neurons in a targeted or untargeted manner to express a range of light-gated ion channels, most commonly the channelrhodopsin-2 cation channel (ChR2) and its variants (Mohanty and Lakshminarayananan, 2015), Figure 3A. ChR2 was originally isolated from the photosensitive algae *Chlamydomonas reinhardtii*, and exhibits a similar retinal moiety response to that found in mammalian photoreceptors capable of eliciting strong depolarizations (Nagel et al., 2003). Inhibitory opsins also exist in the form of light-sensitive ion pumps in the halorhodopsin (Han and Boyden, 2007; Zhang et al., 2007) and archaerhodopsin classes (Chow et al., 2010). Together these offer a flexible and tractable method of neural control which may be used for either fundamental neuroscience research, or as a tool for therapeutic modulation of sensory neurons for prosthesis applications.

**Figure 3:**
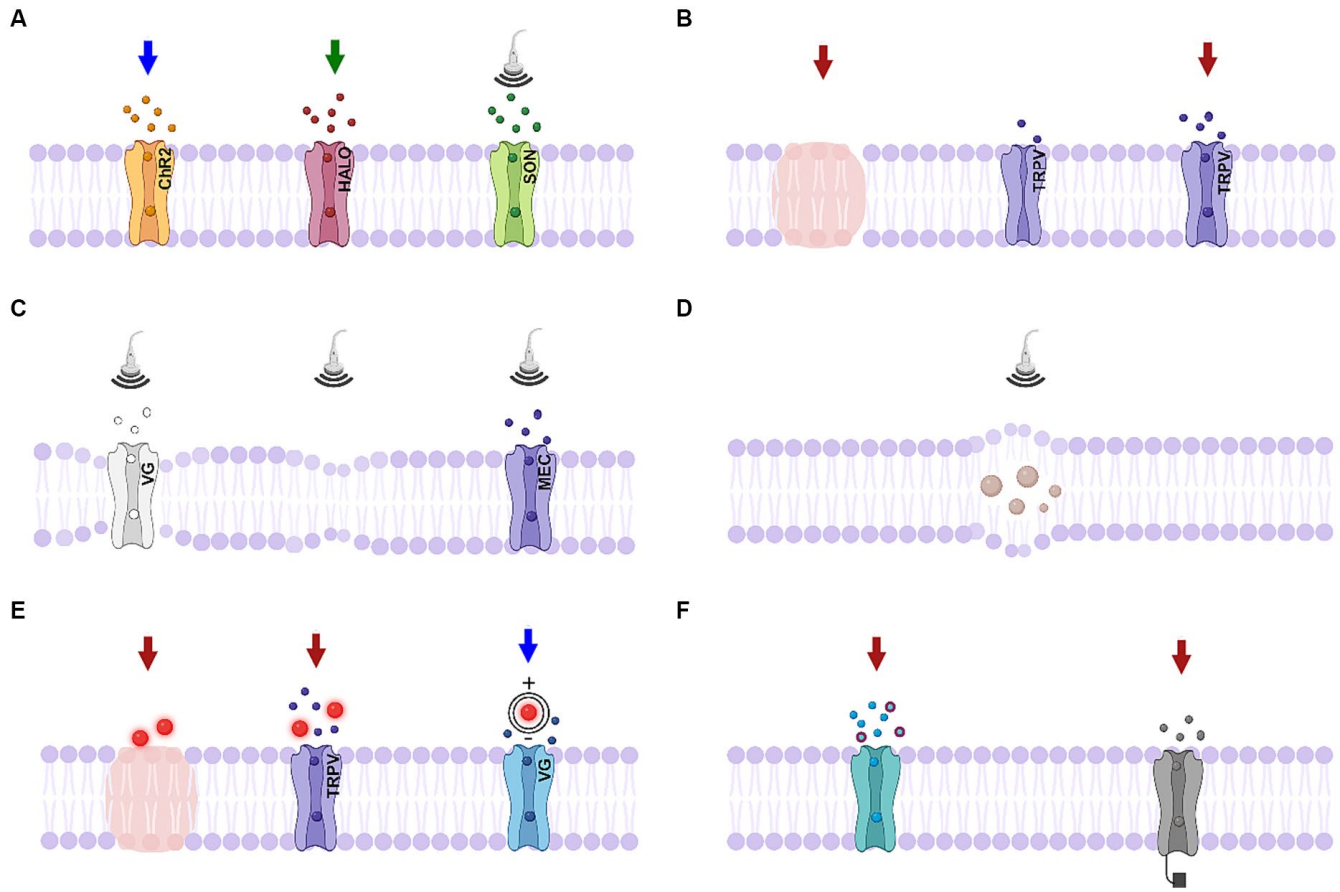
Schematics of biophysical mechanisms for optical and ultrasonic modulation. **(A)** Activation of genetically-modified channels. Left, middle: activation of light-sensitive channels channelrhodopsin-2 (ChR2) and halorhodopsin (HALO); right: activation of ultrasound sensitive proteins (SON, includes TRPV1 ion channels, mechanosensitive ion channel of large conductance – MscL, or auditory-sensing protein prestin). **(B)** Infrared neural modulation. Left: localized heating; middle: closed thermally-mediated TRPV channel; right: activation of thermally-mediated TRPV channel. **(C)** Ultrasonic stimulation. Left: Change in the membrane conformational state causes activation of voltage-gated channel; middle: thermodynamic waves; right: activation of mechanosensitive channel. **(D)** Ultrasonic stimulation causing cavitation effects. **(E)**. Nanoparticle-mediated stimulation. Left: localized heating; middle: activation of TRPV channels; right: nanoparticles generating a dipole moment. **(F)** Photochemical tools. Left: photocaged neurotransmitters; right: photoswitches responding to a particular wavelength of light. Created in BioRender.com (2023).

Importantly, optogenetics is also mutation-independent, whereas inherited retinal diseases are associated with mutations in more than 100 genes, which makes gene-by-gene precision medicine-based approaches challenging (Chew and Iannaccone, 2023; John et al., 2023). Indeed, there is only one currently approved gene replacement therapy for a single form of retinitis pigmentosa caused by a mutation in the gene RPE65 (Russell et al., 2017). In contrast, optogenetics has already been used in retinal-degenerative (rd1 and rd10) mouse models to confer light sensitivity to extant RGCs through ChR2 expression (Zhang et al., 2009; Mohanty and Lakshminarayananan, 2015), and has been shown to restore functional responses to light in non-human primates (Douar et al., 2016; Gauvain et al., 2021).

Several clinical trials using viral vectors have been approved, with positive initial results being reported for safety and efficacy (Sahel et al., 2021; Yan B. Y. et al., 2023). The results to date suggest that the combination of optogenetic treatment together with light-stimulating goggles can provide a level of visual recovery that delivers a rudimentary form of vision (Sahel et al., 2021). Further progress is needed to restore vision to a level that offers the space and time resolution necessary for basic daily tasks. This requires optimization of optogenetic tools and protocols for their safe and reliable delivery at appropriate levels to the most appropriate cell types and cellular sites (Simunovic et al., 2019; Lindner et al., 2022; Stefanov and Flannery, 2023). For example, it has been shown that antagonistic center-surround receptive field interactions can be mimicked by localizing a hyperpolarizing opsin to the dendritic tips and a depolarizing channel to the soma (Greenberg et al., 2011; Wu et al., 2013).

Whilst the challenge is partly to harness the advantages of cell specific targeting to achieve improved spatial resolution, currently available opsins might also face limitations in terms of temporal bandwidth. RGC time constants are on the order of up to 50 ms (Wong et al., 2012), whereas the highest robust reported stimulation frequency for transfected retinae during *in vivo* recordings in non-human primates is 16 Hz (Chaffiol et al., 2022). This is far below the maximum of around 100 Hz reported in electrically-stimulated RGCs (Uzzell and Chichilnisky, 2004). Higher stimulation frequencies may be achievable using hybrid optogenetic-electrical stimulation strategies as reported in the cochlea (Hart et al., 2020), but these have not yet been explored in RGCs.

While there are ongoing concerns about potential long-term complications of optogenetic therapies (Harris and Gilbert, 2022), the clinical trials currently under way are likely to provide important insights into the remodeling of the degenerate retina and challenges of coding visual information at different levels of the extant retinal circuitry. This information is required to improve the clinical outcomes of optogenetic therapies and may also assist in the development of other emerging techniques that allow cell specific targeting.

### 3.2 Infrared neural modulation

As an alternative to optogenetics, infrared light pulses in the wavelength range 1,400–2,200 nm can be used to stimulate and inhibit neurons without any genetic or chemical tissue modifications (Thompson et al., 2014). This technique, termed infrared neural modulation (INM), was first demonstrated *in vivo* with rat sciatic nerve by Wells et al. (2005) Action potentials in a wide range of neural targets has since been observed, including rodent cochlea, cavernous nerves, facial nerves, dorsal root ganglia, cardiac tissue, cortical neurons, and various cultured neurons. Whilst the lack of exogenous material requirements offers significant theoretical benefit in terms of clinical translatability, INM also carries a high risk of thermal damage and exhibits a relatively low penetration depth.

The underlying mechanisms of INM have been shown to rely on the thermal transients generated by the localized absorption of light by water molecules in the target tissue (Wells et al., 2007a; Shapiro et al., 2012), as shown in Figure 3B. It has been observed that the small depolarizing currents evoked by a 1890 nm laser in Xenopus oocytes are similar to the predictions of a conventional capacitance model (Shapiro et al., 2012). These currents exhibited a temperature gradient (dT/dt) dependence rather than scaling with absolute temperature changes (ΔT) and were attributed to thermally-evoked dimensional changes in the neuron’s lipid bilayer. A relatively simple Gouy-Chapman-Stern theory of membrane capacitance was found to accurately predict the main features of the effect and a compelling description of the underlying membrane deformation mechanics has been posited by Plaksin et al. (2018).

Temperature-gated ion channels in the TRPV family may also play a role in INM. Albert et al. (2012) identified TRPV currents as the dominant stimulation mechanism in cultured RGCs and vestibular ganglion cells exposed to 1875 nm light. They were able to evoke action potentials without prior electrical depolarization but noted that this mechanism was highly cell-specific, being present only in neural populations with TRPV expression. In RGCs, TRPV4 was found to contribute significantly to the generation of action potentials, and is temperature gated at around 27–35°C (Watanabe et al., 2002; Caterina, 2007). This relatively wide (8°C) temperature threshold is unique to TRPV4 (Güler et al., 2002), and may be due to a temperature adaptation mechanism under steady-state conditions. Given that TRPV4 gating starts just above room temperature, the ΔT required to elicit action potentials at physiological temperatures may differ significantly from *in vitro* RGC thresholds.

Several researchers have also reported the presence of intracellular calcium transients in response to infrared light (Dittami et al., 2011; Lumbreras et al., 2014; Golovynska et al., 2019). This appears to be the result of thermally-mediated modulation of mitochondrial calcium cycling (Paviolo et al., 2014), although a non-thermal origin has also been proposed (Golovynska et al., 2019). It is currently unclear what (if any) relation intracellular calcium release has to membrane depolarization, but numerous potential mechanisms have been suggested, most notably synaptic vesicle release (Liu et al., 2014; Entwisle et al., 2016).

While transient changes in temperature are effective in stimulating neuronal cells, more sustained rises in temperature (ΔT) have been shown to inhibit activity. Temperature-dependent inhibition was first observed in the giant squid axon by Hodgkin and Katz (1949), who reported faster sodium and potassium voltage-gated ion channel dynamics and a reduced action potential amplitude as temperature increased, which lead to a total inhibition of action potential propagation above 38°C. Huxley postulated that this was due to an increase in potassium conductance at higher temperatures which suppressed sodium-driven depolarization, resulting in action potential block (Huxley, 1959). Recent modeling by Ganguly et al. (2019) appears to support this hypothesis, finding that potassium current hyperpolarization was sufficient to explain action potential propagation in an unmyelinated giant squid axon.

Nanoporation of neural membranes in response to infrared light has also been reported (Beier et al., 2014). There is some limited evidence to suggest that this may take place near the temperature of lipid phase transition, and may thus be a reversible process (Gallaher et al., 2010). Whilst such a phenomenon would certainly be capable of eliciting action potentials, a definitive link between nanoporation and INM has yet to be established.

Intuitively, the thermal nature of INM must place target neurons at some risk of thermal damage (Izzo et al., 2006, 2007; Wells et al., 2007b; Rajguru et al., 2010; Goyal et al., 2012; Matic et al., 2013; Chernov et al., 2014; Liljemalm and Nyberg, 2014; Brown et al., 2020). Conventional INM typically involves laser-evoked heating approaching ∼60°C for durations generally less than a millisecond (Thompson et al., 2014). Whilst many researchers have reported that INM processes are sustainably reversible, the modulation threshold is close enough to the damage threshold to have attracted sustained attention (Wells et al., 2007b; Goyal et al., 2012; Chernov et al., 2014; Liljemalm and Nyberg, 2014; Brown et al., 2020). Depending on the duration for which an elevated temperature is maintained, neurons heated up to ∼42–55°C (hyperthermic) typically exhibit conformational changes in membrane-associated proteins, deactivation of enzymes as well as phospholipid phase changes and membrane rupture (Thomsen, 1991; Welch and van Gemert, 2011). Thermal damage is often modeled as a first-order rate process in which the damage is exponentially dependent on temperature but linearly dependent on the time of exposure (Thomsen and Pearce, 2011). Consequently it is relatively difficult to identify a critical time point for chronic exposure to low-temperature hyperthermic conditions. Depending on the technique used, changes in cells and tissue due to low-temperature thermal injury can only be observed several minutes to a few hours after heating and must be fairly extensive to be distinguished from fixation artifacts (Thomsen, 1991). There is also evidence to suggest that cellular temperature is not uniform throughout the cell. In particular, there have been claims that components such as the mitochondria operate at temperatures of 48°C or more (Chrétien et al., 2018), suggesting that cells may be capable of tolerating relatively high temperatures under certain targeted heating conditions, although this has been disputed (Moreno-Loshuertos et al., 2023).

Since INM relies on infrared wavelengths that are absorbed by water and the eye is highly hydrated, an external light source that has sufficient power to reach the retina would exceed the damage threshold at the cornea. This makes the technique unsuitable for minimally-invasive vision restoration. Nevertheless, the underlying thermal mechanisms and damage considerations are relevant to the following discussion of nanoparticle-based photothermal approaches.

### 3.3 Ultrasonic neuromodulation

The capacity of low intensity focused ultrasound to provide non-invasive and reversible modulation of neural activity with spatial selectivity on the-millimeter scale has been known since 1929 (Harvey, 1929). The technique has recently attracted significant attention for transcranial modulation of the central nervous system, particularly since it was recognized that nerve activity could be excited or suppressed, depending on the combination of ultrasound parameters, experimental models and conditions (Naor et al., 2016; Fomenko et al., 2018; Blackmore et al., 2019; Rabut et al., 2020; Wang et al., 2020; Zhang T. T. et al., 2021; Badadhe et al., 2022). High intensity focused ultrasound is understood to ablate tissue due to heat generated by the mechanical wave interacting with the tissue, whereas low intensity ultrasound has demonstrated a favorable safety profile (Pasquinelli et al., 2019; Radjenovic et al., 2022). However, despite a growing literature on the parameter dependence of the excitatory and suppressive effects of low intensity ultrasound, the underlying mechanisms of action are not yet fully understood (Kamimura et al., 2020; Dell'Italia et al., 2022). Illustrating the complexity, Feng et al. (2019) discuss potential differences between results reported in the central versus peripheral nervous systems, while a systematic review of transcranial ultrasound by Kim et al. (2021) found that only inhibitory effects had been reported in primates and humans.

A wide variety of mechanisms have been proposed and could plausibly contribute to the observed effects in a particular experiment with varying relative importance. These include pressure-induced changes in membrane conformational states due to displacement or bending (Jerusalem et al., 2019), flexoelectricity due to symmetry breaking, generation of thermodynamic waves (solitons) with lossless propagation, triggering of mechanosensitive ion channels (the TRPV channels mentioned in Section 3.2 are both thermo- and mechano-sensitive), vibrational resonances in microtubules that modulate synapses, and intramembrane cavitation (Kamimura et al., 2020; Dell'Italia et al., 2022), Figures 3C,D. The latter mechanism is supported by a comprehensive model (Plaksin et al., 2016) that was able to fit a wide range of experimental observations for multiple types of excitatory cortical neurons, inhibitory neurons and thalamic neurons. This approach illustrates the importance of a unifying hypothesis for ultrasonic neuromodulation to support the design of advanced waveforms that allow cell-type-selective network control.

While conventional ultrasonic transducers, with their need for intimate body contact, are not an obviously advantageous candidate for a visual prosthesis, ultrasound is already widely used for diagnostic imaging of the retina. This confluence has encouraged pilot studies of ultrasonic stimulation of the retina as an alternative approach to vision restoration in cases of retinal degeneration. This topic has been comprehensively reviewed, together with the associated engineering challenges for developing an acoustic retinal prosthesis (Naor et al., 2016; Lo et al., 2020; Rabut et al., 2020; Badadhe et al., 2022; Lu et al., 2022). In an initial report, Naor et al. (2012) were able to record potentials from the scalps of anesthetized rats evoked by ultrasound directed towards their retina. Responses were found to bursts of 0.5 and 1 MHz ultrasound, with peak acoustic pressures ranging from 86 to 725 kPa, total durations of 5–20 ms and pulse repetition frequency on the order of 2 kHz. This work also identified a need to generate continuous ultrasound patterns, proposing a holographic multifocal approach, where the required pattern is created by interference between the waves emanating from multiple sources on a phased array. Analysis of trade-offs between ultrasound frequency and resolution, and frequency and safe intensity, suggest that approximately 2,500 pixels could be stimulated across the retina at 2.5 MHz. Notably, OFF responses were also observed in single RGCs in isolated mouse retina in response to continuous wave 2.3 MHz ultrasonic stimuli from a clinical phased array transducer (Naor et al., 2016).

Subsequently, Menz et al. (2013) compared visual stimulation of RGCs in the isolated salamander retina to stimulation with high frequency (43 MHz) ultrasound. They found repeatable spike responses to both ultrasound onset and offset, and that increasing intensity led to higher firing rates and decreased latencies (saturating at 10–30 W cm^−2^). Only stimulus frequencies within the “physiological” range (<15 Hz) affected neural activity, whereas the response was insensitive to changes in the pulse repetition rate from 15 Hz up to 1 MHz. The tight 90 μm focal spot enabled the observation of phenomena similar to the visual center-surround antagonism, which indicates processing within the retinal network and implies that the ultrasound in part stimulated cells other than ganglion cells directly. Pharmacological manipulations indicated only a minor contribution of photoreceptors in the process, and a critical role of Ca^2+^ currents.

More recently, it has been shown that ultrasonic stimulation can reliably activate the degenerative retina of both normal sighted and Royal College of Surgeons (RCS) rats, which are a widely recognized model of retinitis pigmentosa (Gal et al., 2000). Ultrasound activation was observed *in vivo* with a high spatiotemporal resolution, while visible light stimulation failed to show any retinal responses in the RCS rat (Qian et al., 2022). Ultrasound-evoked neuronal activity was measured *in vivo* by means of electrodes in the contralateral visual pathways including the superior colliculus and the visual cortex of the brain, in either normal-sighted or RCS rats. Neuronal activity induced by a spherically focused 3.1 MHz ultrasound transducer demonstrated spatial resolution of 250 μm and temporal resolution of 5 Hz in the rat visual centers. Based on the retinotopic properties of the superior colliculus, the investigators were also able to decode the static stimulation pattern of letter forms induced by ultrasound in the retina.

These demonstrations of retinal stimulation have been accompanied by some proposals for miniaturized transducers. Gao et al. (2017) described a contact-lens array transducer concept for use in an ultrasound retinal prosthesis that could be acoustically coupled to the eye via the tear film. Similarly, a ring array transducer with a hemisphere surface has been proposed that mimics a contact lens and could be used to acoustically couple with the eye via the tear film. This design would avoid the high acoustic absorption associated with the crystalline lens by directing the ultrasound around the lens (Xu et al., 2022).

Another interesting alternative is so-called sonogenetic therapy, which is an extension of the optogenetic techniques discussed in Section 3.1. Sonogenetics relies on the activation of mechanosensitive channels by means of ultrasound waves that can penetrate the dura mater and brain tissue (Wang et al., 2020; Provansal et al., 2022; Cadoni et al., 2023). It has also been used to enhance the sensitivity to low intensity focused ultrasound in the retina and the visual cortex (Cadoni et al., 2023). Wild-type and G22S mutation of the mechanosensitive ion channel of large conductance (MscL) were targeted to rat RGCs by intravitreal delivery of an adeno-associated vector. With this sensitization, an unusually high-frequency 15 MHz ultrasonic stimulation could be used to activate retinal neurons with millisecond temporal precision and a spatial resolution of 0.06 mm. The worst-case spatial peak temporal average power intensities of 1.56 W cm^−2^ for repeated stimulations at 13 Hz rate were similar to those that are safely used in clinical diagnostic imaging. Modulation of the visual cortex suggests the potential for vision restoration via a brain-machine interface.

## 4 Nanophotonic transduction mechanisms

All the less invasive neuromodulation modalities introduced above can be extended or modified by the addition of functional nanoparticles. Nanoparticles are generally considered to have dimensions of 100 nm or less. However, the definition is necessarily imprecise, as these materials are also characterized by unusual physical, chemical and biological properties that are not present in the bulk material. These properties can be leveraged to improve the efficiency of energy transduction into the biological tissue. The small size of nanoparticles can also serve to improve the spatial and/or temporal resolution of the intervention. This implies the potential to reduce off-target effects and enhance cell-type selectivity, especially in combination with surface chemical modifications of the nanoparticles that can be used to target specific cell markers. This section introduces a range of nanoparticle transducers that can be addressed by light and have been (or could be) used in the context of retinal neuromodulation. Some interesting examples of magnetically- and acoustically-actuated nanoparticles are briefly discussed in Section 5. For convenience, the key works in this domain have been summarized in Table 1, together with some examples of nanoparticles that have been reported for drug delivery or other therapeutic effects in the retina.

**Table 1:** Summary of nanoparticles that have been used to modulate retinal activity, deliver drugs to the retina, or for therapeutic effects in the retina.

Nanoparticle	Size	Irradiation	Material/Functionalization	Mechanism	Application	Delivery, observed effects
Au NRs with ectopically expressed TRP channels	75 × 20 nm 115 × 20 nm	915, 980 nm; 100, 200 ms; 0.2–2 W cm^−2^	6x-His antibodies	Photothermal	Restoring light sensitivity in blind mice	Subretinal injection of 10^10^ NRs per eye. Light-evoked responses in mouse primary visual cortex, light–guided mouse behavior, responses in *ex vivo* human retina (Nelidova et al., 2020)
Au NRs	52 × 14 nm	780 nm; 0.1, 0.5, 200 ms; 2–7 kW cm^−2^	Streptavidin coated	Photothermal	Retinal neuromodulation	Sedimentation on explanted rat retina in culture medium. Stimulation of RGCs for sub-ms laser pulses, inhibition of RGCs for 200 ms pulses (Begeng et al., 2023)
Au nanospheres	20 nm	N/A	Not specified	Passive diffusion	Drug delivery through blood-retina barrier	Intravenous injection at 1 g kg^−1^ in mice. Nanospheres distributed into all retinal layers, including neurons, endothelial cells and peri-endothelial glial cells. No observed toxicity (Kim J. H. et al., 2009)
Au nanospheres	100 nm	800 nm; 100 fs pulses for 15 s; 1.5–8.8 kW cm^−2^	K_V_1.1 antibodies	Photothermal	RGC optoporation for drug delivery	Intravitreal injection of 5 μL in rat eyes. Targeted drug delivery to RGCs without cell death (Wilson et al., 2018)
P3HT	300 nm	White light 400–700 nm; 200–500 ms; 1–10 W cm^−2^	poly[3-hexylthiophene]	Photovoltaic	Rescuing visual function in blind rats	Intravitreal injection of 10–20 μL in rat eyes. Wide retina coverage and retention at 240 days post injection. Stimulation of visually-evoked potentials *in vivo*, light driven behaviors (Maya-Vetencourt et al., 2020; Francia et al., 2022)
DENAQ/BENAQ	N/A	White light 400–550 nm; <0.2 mW cm^−2^	Formate salt	Photoswitch	Restoring visual function in blind mice	Intravitreal injection of 2 μL in mouse eyes. Restored electrophysiological and behavioral responses with no toxicity (Tochitsky et al., 2014, 2017)
Upconversion nanoparticles	38 nm	980 nm LED; ~160 ms; 1.62 mW cm^−2^	Concanavalin A conjugated poly acrylic acid-coated β-NaYF_4_:20%Yb, 2%Er@β-NaYF_4_ core-shell particles	Photon upconversion	Near-infrared vision	Sub-retinal injection of 2 μL in mouse eyes. Near infrared light patterns were perceived, based on single-photoreceptor recordings, electroretinograms, cortical recordings, visual behavioral tests (Ma et al., 2019)
Polystyrene, PLGA and DNA nanoparticles	100–1,000 nm	N/A	Amine-, carboxyl- and PEG-modified polystyrene, CK_30_PEG_10k_/DNA, PVA-coated PLGA	Passive diffusion	Drug delivery to sub-retinal space and RPE	Intravitreal injection of 2–5 μL in *ex vivo* bovine eyes. Anionic particles diffused through vitreous meshwork but cationic particles were immobilized. Average mesh size estimated to be 550 nm (Xu et al., 2013)
Human serum albumin nanoparticles	107–176 nm	N/A	Hexamethylenediamine (cationic particles), Alexa555 (anionic particles)	Passive diffusion	Drug delivery to sub-retinal space and RPE	Intravitreal injection of 2 μL in rat eyes. Faster diffusion of anionic particles. Taken up by Müller cells and reached choroidal space (Kim H. et al., 2009)
Polymeric nanoparticles	230–345 nm	N/A	Polyethyleneimine (PEI), glycol chitosan (GC), hyaluronic acid (HA), human serum albumin (HSA), PEI/GC, HSA/GC and HSA/HA nanoparticles	Passive diffusion	Drug delivery to sub-retinal space	Intravitreal injection of 5 μL in rat eyes. Anionic HA and HSA nanoparticles penetrated across the whole retina to the RPE via Müller cells (Koo et al., 2012)
Core-shell liponanoparticles	320 nm	N/A	Hyaluronan modified, RITC labeled	Passive diffusion	Drug delivery to sub-retinal space and RPE	Intravitreal injection of 10 μL in rat eyes. Targeted CD44 on RPE cells with 75% of particles retained in RPE or choroid after 7 days (Gan et al., 2013)
Cerium oxide nanoparticles	3–5 nm	N/A	Vacancy-engineered mixed-valence-state cerium oxide	Catalytic activity	Antioxidant treatment of age-related macular degeneration, inherited retinal degeneration	Intravitreal injection of 2 μL in rat eyes. Prevented loss of vision due to light-induced degeneration of photoreceptor cells (Chen et al., 2006)
Magnetite nanoparticles	17–252 nm	N/A	glucuronic acid (anionic), polyacrylic acid (anionic), polyethyleneimine (cationic), polyglucose sorbitol carboxymethylether (neutral)	Passive diffusion	Drug delivery to sub-retinal space and RPE	Intravitreal injection of 70 ng in *Xenopus* embryos and zebrafish. Nanoparticles localized specifically in RPE and were retained for 20 days. No observed toxicity (Giannaccini et al., 2014)
Liposomes	120 μm	Blue Ar^+^ laser; 0.1–0.5 s pulses; “eye safe“	Dipalmitoylphosphatidylcholine, dipalmitoylphosphatidylglycerol	Photothermal	Drug delivery in the ocular vasculature	Intravenous injection of rats. Heating due to absorption of light by blood and melanin led to dye release at 41°C in choriocapillaris (Zeimer and Goldberg, 2001)

Where relevant, the irradiation parameters include wavelengths or wavelength range, pulse lengths and irradiances (power per unit area). The tabulated irradiances refer to ranges reported as effective and safe within the context of the respective studies, but values above ~1 kW cm^−2^ may exceed the established guidelines on limits of exposure to laser radiation [Delori et al., 2007; International Commission on Non-Ionizing Radiation Protection (ICNIRP), 2013]. In some cases, the mechanism may include more than one effect, or may not be fully understood.

### 4.1 Photothermal effects

Optically-absorbing nanoparticles have seen increasing use in INM applications as a replacement for heating via water-absorption (Paviolo and Stoddart, 2017; Hart et al., 2019). Nanoparticle-enhanced infrared neural modulation (NINM) places thermally-transducing nanoparticles around a neural target and applies targeted illumination to elicit rapid nanoscale heating at the particle surface, as shown in Figure 3E. The earliest report of this approach used visible and near infrared light to illuminate black photo-absorbing micro-particles (diameter ~ 6 μm) and stimulate nearby cortical neurons (Farah et al., 2013). Nanoparticles synthesized from metals such as silver and gold have a high number of mobile electrons and are efficient at converting optical energy to heat. Optical absorption generates electron charge oscillations called surface plasmons, which in nanoparticles are strongly confined by the particle’s small surface dimensions (i.e., the boundary conditions) (Govorov and Richardson, 2007; Richardson et al., 2009). For a given incident photon wavelength, there then exists a ratio between the length and width (aspect ratio) of a planar surface that produces a resonance effect in the surface plasmon wave, known as the localized surface plasmon resonance (LSPR). Incident light at the LSPR wavelength is absorbed with high efficiency and dissipates its energy almost entirely into heat through electron–electron and electron–phonon coupling (Richardson et al., 2009).

Spherical nanoparticles have an aspect ratio of one, whilst gold nanorods possess an additional resonance tunable to the ratio between their length and diameter (Paviolo and Stoddart, 2017). Because the longitudinal mode of the gold nanorods can be precisely tuned during particle synthesis, the stimulus wavelength can be selected arbitrarily. Therefore, most NINM techniques take advantage of the near-infrared optical window in biological tissue for which optical absorption is low, defined by the simultaneously low water and hemoglobin absorption coefficients (600–1,400 nm) (Eom et al., 2014; Yong et al., 2014; Yoo et al., 2014). In this wavelength range optical scattering and power losses are relatively small (Hale and Querry, 1973), allowing for efficient heating at higher penetration depths. In the retina, these wavelengths should also serve to avoid spurious responses due to residual photoreceptor activity.

NINM has been applied in a wide range of *in vitro* and *in vivo* targets, with stimulatory effects reported in hippocampal neurons (Lavoie-Cardinal et al., 2016; Lee et al., 2018), dorsal root ganglions (Carvalho-de-Souza et al., 2015, 2018), bullfrog and rat sciatic nerves (Eom et al., 2014; You and Mou, 2017; Mou et al., 2018), spiral ganglion neurons (Yong et al., 2014), HEK-293 cells (Martino et al., 2015), cortical neurons (Farah et al., 2013; Johannsmeier et al., 2018), NG-108-15 cells (Paviolo et al., 2014) and rat astrocytes (Eom et al., 2017). Similar to the inhibitory effect observed in INM (Section 3.2), nanoparticle-mediated photothermal processes have also been shown to inhibit action potential generation and propagation in a wide range of neurons including rat cardiomyocytes (Wang et al., 2016), primary hippocampal neurons (Yoo et al., 2014; Lee et al., 2018; Xia and Nyberg, 2018) and *Aplysia californica* motor neurons (Duke et al., 2013; Lothet et al., 2017).

More recently, Nelidova et al. (2020) demonstrated substantial vision rescue in awake rodent models by optical stimulation of subretinally-injected plasmonic nanoparticles. In this modified NINM approach the GNRs were targeted to thermosensitive TRPV1 ion channels in the mouse photoreceptors. This required genetically modification of the photoreceptors, as TRPV1 channels do not occur in the wild-type murine retina. These results suggest that NINM has potential to be a safe alternative to existing retinal prosthesis technologies, although the strategy of targeting nanoparticles to photoreceptors may be not suited for late-stage retinal degeneration, which corresponds to the atrophy of rods and cones (see Section 2). As a generalization of this approach, our lab has subsequently demonstrated that NINM can be applied directly to RGCs in explanted rat retinae, without the need for genetic modification (Begeng et al., 2023). We found that shorter (sub-millisecond) laser pulses evoked robust RGC stimulation by capacitive current generation, while longer 200 ms laser pulses were capable of inhibiting spontaneous action potentials by thermal block.

Using gold nanorods and carbon-based micro-particles in close proximity to dorsal root ganglion neurons, Carvalho-de-Souza et al. (2018) demonstrated the potential efficiency gains of NINM. They were able to elicit action potentials from 785 nm pulses with energies as low as 76 nJ (from a 1 μs pulse). This surprisingly low threshold is a result of the highly-localized nature of nanoparticle heating, which can be in excess of 10°C at the particle surface but decay to negligible values at several micrometers displacement (Eom et al., 2018). Moreover, Carvalho de Souza’s work highlights a key implication of the capacitance mechanism identified by Shapiro et al. (2012) (see Section 3.2): if capacitive currents depend only on temperature gradient dT/dt, the maximum evoked ΔT can be made arbitrarily small. In other words, a laser spot with sufficiently high irradiance and a short pulse duration can theoretically evoke action potentials with negligible total heating. Indeed, a recent modeling study by Eom et al. (2018) estimated that localized gold nanorod heating of less than 3°C would be sufficient to elicit action potentials at an irradiance of 240 W cm^−2^, even in the absence of a TRPV current.

In contrast to this thermally-evoked capacitive current mechanism, infrared-evoked inhibition appears to be due to an absolute increase in temperature, rather than a temperature gradient (Duke et al., 2013). There is strong experimental evidence to suggest that very high frequency biphasic electrical stimulation may be capable of inducing depolarization block in RGCs (Twyford et al., 2014; Kameneva et al., 2016). With that exception, the less invasive techniques discussed in Section 3 may offer advantages in terms of suppressing spontaneous RGC activity. The successful demonstration of safe thermal block with NINM in RGCs by Begeng et al. (2023) shows promise for the development of nanoparticle-based retinal interfaces that combine stimulatory and inhibitory inputs to achieve a high visual acuity. However, by requiring exogenous nanoparticle absorbers, NINM forgoes a key advantage of conventional INM. On the other hand, GNRs confer an additional benefit in their versatile choice of coatings, which can be selected to target specific cell populations. Specific antibodies can be selected to target a range of neuronal membrane proteins, including voltage-gated sodium channels, TRPV1 ion channels and P2X_3_ receptor ion channels (Carvalho-de-Souza et al., 2015), or even be cholesterol-coated for nonspecific membrane integration (Carvalho-de-Souza et al., 2019). P2X_3_ purinoceptors are involved in fast, excitatory neurotransmission in the nervous system, and are expressed predominantly within sensory neurons. In the rat retina, P2X_3_ receptors have been shown to be most commonly expressed in the inner plexiform layer, which lies immediately below the ganglion cell layer (Puthussery and Fletcher, 2007). Gold nanoparticles coated in Kv1.1 antibodies have also been used to target RGCs as contrast agents for optoporation and subsequent drug delivery in the rat retina (Wilson et al., 2018).

As discussed in the context of conventional INM (Section 3.2), excessive levels of optically-induced heating can have a number of deleterious effects. It is interesting to note that many of these disruptive phenomena have also been observed for nanoparticle-mediated heating, including reversible changes in membrane conductance (Urban et al., 2016), formation of vapor bubbles and cell poration (Pitsillides et al., 2003; Yao et al., 2005), and cell death (Hirsch et al., 2003; Palankar et al., 2014). These effects have been well documented and become more pronounced for shorter laser pulses (ps to fs) that can reach higher peak powers (Boulais et al., 2013). In general, NINM techniques aim to avoid these effects by staying well below the damage threshold, but under certain conditions the high degree of energy localization around the nanoparticles may also lead to useful mechanical or acoustic effects that are discussed in the following section. It is also important to note that nanoparticle concentration plays an important role in the apparent heating effects: for a high concentration of NPs and small laser intensity, the bulk temperature increase is significant compared to the temperature increase at the surface of a single nanoparticle, whereas for a small NP concentration and intense laser irradiation, there is a small temperature increase in the bulk with strong nanoscale temperature spikes at the NP surfaces (Richardson et al., 2009). This highly localized nanoscale heating effect may serve to ameliorate the severe limitations predicted for conventional INM at dense modulation site spacing and high pulse repetition rates (Thompson et al., 2013).

### 4.2 Photomechanical and photoacoustic effects

Pressure waves generated by conventional ultrasound transducers have been exploited to stimulate nerves, either directly (Section 3.3) or indirectly via interactions with piezoelectric nanoparticles (Marino et al., 2015; Cafarelli et al., 2021) or by inducing currents through interaction with an applied magnetic field (Norton, 2003). Similarly, optically-responsive nanoparticles can give rise to a range of photomechanical or photoacoustic effects, depending on the type of nanoparticle, the type of target cell, the location of the nanoparticle relative to the target cell, and the type of optical input (Kolb et al., 1995; Willermain et al., 2014).

For laser pulses below the ablation threshold in soft tissue, the confinement of laser energy in both space and time has a well understood dependence on laser penetration depth and pulse duration (Jacques, 1992). In the absence of confinement effects, the laser heating can be thought of as a conventional heat source that generates temperature changes consistent with thermal diffusion. This corresponds to the situation observed for high concentrations of nanoparticle absorbers and low laser intensities, as discussed in the previous section (also see Richardson et al., 2009). For shorter pulses and/or longer penetration depths that correspond to lower nanoparticle concentrations, if the laser pulse width is much shorter than the thermal relaxation time, then the thermal energy remains confined within the absorption volume during the laser pulse. This “thermal confinement” occurs when the laser pulse duration is less than $\delta^{2}/4\kappa$ but greater than $\delta /v_{s}$ , where *δ* is the laser penetration depth (or the size of the absorbing structure), *κ* is the thermal diffusivity of irradiated material and *v_s_* is the speed of sound in the medium. In this regime the effects of thermal diffusion are negligible on the timescale of the laser pulse and typically corresponds to microseconds to millisecond pulses for near infrared wavelengths in tissue. Thermal confinement is associated with infrared neural stimulation (Wells et al., 2007a) (Section 3.2) and the photothermal stimulation effects described in Section 4.1.

For laser pulses shorter than $\delta /v_{s}$ , the thermoelastic expansion of the tissue due to light absorption leads to propagating pressure or stress waves, so this regime is referred to as “stress confinement.” The capacity of nanoparticles to generate acoustic waves has been known for some time, with applications in drug delivery (Zhang et al., 2017; Wang J. B. et al., 2018), and as contrast agents in photoacoustic (and photothermal) imaging (Wu et al., 2019; Steinbrueck and Karges, 2023), or as combined modalities in theranostics (Qin et al., 2023; Yan T. J. et al., 2023).

In the context of neuromodulation, a photoacoustic modality stands to benefit from the advantages of ultrasound with low levels of heating, as well as the high spatial precision of photons. Shi et al. (2022) have reviewed recent developments in a variety of photoacoustic platforms for neural modulation. To date, the only colloidal nanoparticles that appear to have been reported as an absorption agent for photoacoustic neuromodulation appears to be semiconducting polymer nanoparticles based on bis-isoindigo (BTII) modified with poly(styrene)-b-poly(acrylic acid) (PS-b-PAA) through a nanoprecipitation method to form water-soluble nanoparticles with a size of ∼50 nm (Jiang et al., 2021). These particles strongly absorb in the second near-infrared window, which maximizes tissue penetration. The surface modification of the semiconductor nanoparticles promoted selective binding to neurons and allowed activation of primary neurons *in vitro* by means of ten 3-ns laser pulses at 1,030 nm over a 3-ms duration with a peak pressure of 1.36 kPa. Antibody conjugation of the nanoparticles with mechanosensitive TRPV4 channels significantly increased the stimulation success rate to 53.3% in the presence of synaptic blockers. *In vivo* neural modulation of mouse brain and motor activities was also demonstrated by directly injecting the particles into brain cortex (Jiang et al., 2021). In principle, all the ultrasonic stimulation processes shown in Figures 3C,D could be enhanced by the localized energy transduction of nanoparticle absorbers.

In other broader applications of nanoparticle optical absorption agents, graphite (Jiang et al., 2020), carbon nanotubes (CNTs) (Shi et al., 2021) and candle soot (Chen et al., 2022) have been incorporated on a tapered fiber photoacoustic emitter for high spatial resolution stimulation, while CNTs have also been embedded in silk scaffolds for neural stimulation and regeneration (Zheng et al., 2022). Li et al. (2022) have extended these concepts by embedding candle soot particles in a curved polydimethylsiloxane film to form a soft photoacoustic pad that can generate a transcranial ultrasound focus at 15 MHz with a lateral resolution of 83 μm.

Gold nanoparticles are the most prominent nanomaterial that has been used to mediate photomechanical interactions in cellular biology. If thermal heating of the environment occurs, this may be damaging for the cell, but can be minimized by using laser pulses of less than about 100 ps (Xiong et al., 2016). However, the use of shorter laser pulses with stress confinement raises the risk of cell damage due to non-thermal effects. The main photomechanical effect of plasmonic nanoparticles is the formation of vapor bubbles upon high energy pulsed optical stimulation at the LSPR peak (Lalonde et al., 2013; Vanzha et al., 2016). While intramembrane cavitation has been proposed as a central mechanism for ultrasonic neuromodulation (Section 3.3) (Plaksin et al., 2016), nanoparticles in close proximity to the target cells may generate vapor bubbles that then create transient pores in the cell membrane or organelles to modulate cellular behavior in a process known as optoporation (Lalonde et al., 2013; Vanzha et al., 2016). These pores can arise due to hydrodynamic stress upon expansion of the vapor bubbles or by liquid jets and shockwaves when the bubbles collapse under hydrostatic pressure (Xiong et al., 2016). In addition, pore closure can take up to several minutes (Caprettini et al., 2016), thus exposing the cell to the extracellular environment for an extended period of time. Therefore, vapor bubble formation appears unlikely to be favored for *in vivo* applications such as retinal neuromodulation. However, it may be of interest for drug delivery (see Section 6). For example, Caprettini et al. (2016) have used optoporation to modulate drug intake in the N2A neuronal cell line.

It should also be noted that the boundary between stress confinement and thermal confinement is not distinct. Heat is an unavoidable by-product of all the processes discussed above. Given the known photothermal effects of nanoparticles discussed in Section 4.1, this issue assumes greater importance when interpreting nanoparticle-mediated photoacoustic effects. Drawing on the experience of low intensity ultrasound neuromodulation (see Section 3.3), conventional delivery parameters are expected to produce relatively modest thermal increases ranging from 0.002 to 0.8 °C (Constans et al., 2018), which are unlikely to produce thermally based neuromodulation (Wahab et al., 2012; Plaksin et al., 2014). However, as seen in the previous Section 4.1, nanoparticle-mediated photothermal processes could potentially exceed these conventional expectations and impose thermal modulatory effects (Darrow et al., 2019). Although the evidence to date appears to discount photothermal contributions (Shi et al., 2022), thermal modeling (Section 7) (Constans et al., 2018) and temperature measurement with appropriate spatial and temporal resolution should be employed to account for the different sonication parameters, tissue properties (e.g., density, perfusion, absorption coefficients), and beam/scanning configurations (Dalecki, 2004).

### 4.3 Photovoltaic effects

Given the relatively well-established nature of electrical interfaces for neuromodulation, it is perhaps unsurprising that photovoltaic interfaces have received significant attention. The most developed of these include implantable photodiode arrays activated by pulsed infrared light (Mathieson et al., 2012; Lorach et al., 2015; Ho et al., 2019), which are already nearing clinical application. Recent progress in this regard has demonstrated high-resolution prosthetic vision based on dynamic field confinement in a novel design of a photovoltaic array, which leverages the adjustable conductivity of the diodes under forward bias to turn the designated pixels into transient returns (Wang et al., 2022).

Other approaches have explored the use of polymer-based photovoltaic implants to reduce the mechanical mismatch arising from semiconductor photodiode arrays. Ghezzi et al. (2013) used an organic film of poly(3-hexylthiophene) (P3HT) to restore light sensitivity in explants of rat retinas with light-induced photoreceptor degeneration. This approach has been extended to form a wide-field, high-density and high-resolution polymeric photovoltaic epiretinal prosthesis for artificial vision, which is comprised of 10,498 physically and functionally independent photovoltaic pixels (Chenais et al., 2021). In an alternative approach, a multilayered architecture of ZnO nanoparticles, PbS quantum dots and P3HT layers with individual layer thicknesses of 50, 25, and 50 nm, respectively, were solution processed onto an ITO/polyethylene terephthalate (PET) substrate to form a flexible photovoltaic biointerface (Karatum et al., 2022). The multilayer structure is intended to convert near infrared light to safe capacitive ionic currents. The device was shown to generate reproducible action potentials on primary hippocampal neurons with high success rates.

In contrast, Bareket et al. (2014) conjugated semiconducting CdSe/CdS core/shell nanorods to a neuro-adhesive 3D carbon nanotube surface via a plasma polymerized acrylic acid mid-layer. Light insensitive embryonic chick retinas were successfully stimulated on this platform. While encapsulation of quantum dots or nanorods in polymeric films can temporarily help to stabilize the particles *in vivo*, concerns still remain about the cytotoxicity of these materials when exposed to cell metabolytes (Mancini et al., 2008). As an alternative, oriented gold nanoparticle-decorated titania (Au-TiO_2_) nanowire arrays have been used as artificial photoreceptors in blind mice (Tang et al., 2018). Grown on fluorine-doped tin oxide or flexible polymer substrates, the 1D semiconductor nanowires exhibit good biocompatibility, efficient photoabsorption, and large charge separation and transport mobility, with an orientation and anisotropy that is analogous to the morphology and architecture of photoreceptors. The gold nanoparticles are intended to enhance the photoconversion efficiency of the nanowire arrays into the visible range. Green, blue and near UV light responses in the RGCs were restored with a spatial resolution better than 100 μm in retinal explants. After subretinal implant of the Au-TiO_2_ nanowire arrays, neurons in the primary visual cortex responded to light and recovery of pupillary light reflex was also observed.

In a progression of these efforts, Maya-Vetencourt et al. (2020) showed that P3HT conjugated polymer nanoparticles could be used to mediate light-evoked stimulation of retinal neurons when subretinally injected in RCS rats. The nanoparticles were observed to spread out over the entire subretinal space without triggering trophic or proinflammatory effects for the maximum observed period of 240 days post injection. The recovery of light-dependent responses in the dystrophic rats was demonstrated by an increase in light-evoked pupil constriction over the whole range of irradiances, while visual functions were confirmed at the cortical level by extracellular recordings of VEPs, visual acuity in response to patterned visual stimuli of increasing spatial frequency was restored, and visually driven behavioral activity was rescued. These effects were observed at both 30- and 240-days post injection with respect to either untreated or sham-injected RCS animals. Given that the currents generated by the 300-nm sized particles (~μA cm^−2^) are orders of magnitude lower than the values needed to open voltage-dependent conductances, a capacitive coupling mechanism that relies on the close contact between nanoparticles and the very high electrical resistance of the neuronal membrane was proposed, as illustrated in Figure 3E. In subsequent work, this group has demonstrated that the P3HT conjugated polymer nanoparticles can reinstate physiological signals at the cortical level and visually driven activities when injected in 10-months-old RCS rats with fully light-insensitive retinas. The extent of visual restoration was reported to positively correlate with the nanoparticle density and hybrid contacts with second-order retinal neurons (Francia et al., 2022).

In response to the work of Maya-Vetencourt et al. (2020), it has been observed that the irradiance levels used in *ex vivo* experiments (500 ms exposures at 40 mW mm^−2^ and a wavelength of 540 nm) were higher than the maximum permissible exposure of the retina and are likely to have a thermal effect on the cells if the nanoparticles are efficient absorbers (Palanker et al., 2021). In their response, Benfenati and Lanzani (2021b) claim that the relatively small nanoparticle concentration used in the retinal explants leads to a temperature rise in the mK range, which rules out the contribution of thermal effects. They also reiterate that the efficiency of charge transport between nanoparticles and cells is dependent on establishing a tight interface with the neuronal membrane. However, as discussed in Section 4.1, in the case of a low NP concentration and intense laser irradiation, there could be a small temperature increase in the bulk together with relatively large nanoscale temperature spikes at the NP surfaces (Richardson et al., 2009). We also note that QDs, in addition to being excellent fluorescent probes, can be used as photoacoustic and photothermal contrast agents and sensitizers (Shashkov et al., 2008). As in Section 4.2, these arguments once again suggest a need for more careful measurement and control of temperature over the relevant spatial and temporal scales.

### 4.4 Photochemical and upconversion effects

Nanoparticle-based photochemical processes have been explored for drug delivery in neurological research (Rwei et al., 2015) and neuromodulation (Ellis-Davies, 2007; Kramer et al., 2009; Warther et al., 2010). These tools are seen to offer the potential for remote, bidirectional manipulation with high spatiotemporal precision of the electrical and chemical signals that affect the activity of cells and circuits. Nanoparticles that present photochemical effects typically contain photo-responsive pendant or functional groups that induce a chemical change within the structure. This is most commonly in the form of isomerization, dimerization and bond cleavage reactions, but designer receptors exclusively activated by designer drugs (chemogenetics) and optogenetic techniques have also attracted significant interest (Rogan and Roth, 2011; Peeters et al., 2020).

UV and blue light are generally advantageous for driving photochemical processes, as these wavelengths can induce very rapid structural changes. For the same reason they are more likely to produce cytotoxic products and, in the present context, interfere with residual visual function in the degenerate retina. While near infrared wavelengths can be used to drive drug release via the photothermal response of plasmonic nanoparticles (see Section 4.1), these wavelengths can also be used to generate photochemical responses through two photon events and upconversion processes (Fino et al., 2009; Warther et al., 2010), thus reducing exposure to the phototoxic effects of UV and blue wavelengths of light. Excitation wavelengths in the near infrared tissue window (650–1,350 nm) can also penetrate tissues more deeply. While two-photon processes can be used directly (Ronzitti et al., 2017), upconversion nanoparticles can provide greater conversion efficiencies without the need for sophisticated ultrafast laser sources. Indeed, it has been shown that millisecond single pulse excitation with high peak power can optimize the intrinsic quantum yield of the particles while moderating the thermal side effects (Liu et al., 2013). In the present context, this approach has mainly been used in conjunction with optogenetics (Shah et al., 2015; Chen et al., 2018). Spectrum-selective upconversion nanoparticles have been used for multiplexed optogenetic stimulation by selectively tuning the emission spectra with different doping strategies to match the responsive wavelength of multiple opsins (Lin et al., 2017). In an interesting extension, Ma et al. (2019) developed injectable photoreceptor-binding upconversion nanoparticles that attached to retinal photoreceptors as miniature near infrared light transducers. Based on single-photoreceptor recordings, electroretinograms, cortical recordings, and visual behavioral tests, it was shown that mice injected with these particles could perceive near infrared light patterns.

Photo-responsive nanomaterials and small molecules have been applied for the photochemical control of neuromodulation. For example, bistable molecules have been used to create photoswitches with multiple configurations that can change upon exposure to light of different wavelengths (Banghart et al., 2004; Kramer et al., 2009; Beharry and Woolley, 2011, Figure 3F). This property has given rise to photoswitchable ligands that can control neurons by regulating K+ channels and glutamate receptors (Fortin et al., 2008; Kramer et al., 2009). A chemical photoswitch named DENAQ that confers light sensitivity on voltage-gated ion channels has been used to restore retinal responses to white light of similar intensity to ordinary daylight (Tochitsky et al., 2014). A single intraocular injection of DENAQ in mice with degenerated photoreceptors was sufficient to photosensitize the blind retina for days, restoring electrophysiological and behavioral responses with no toxicity. DENAQ appears to confer light sensitivity on a hyperpolarization-activated inward current that is enhanced in degenerated retina, thereby enabling optical control of retinal ganglion cell firing. However, the RGCs of DENAQ-treated blind mice all generated the same polarity light response. Subsequent improvements to this photoswitch have increased the sensitivity and *in vivo* stability of the compound (Tochitsky et al., 2017). It may be possible to extend this general approach - which is now known as photopharmacology to distinguish it from optogenetics and chemogenetics - by engineering new molecules for modulating a wider range of specific targets (Broichhagen et al., 2015).

Caged glutamate is one of the most commonly used molecules for neural stimulation (Wieboldt et al., 1994; Shoham et al., 2005), but a number of other neurotransmitters have also been used (Ellis-Davies, 2007). Initially, short wavelengths of about 380 nm were required to activate the cages. However, the high level of light-scattering in tissue at these wavelengths tends to limit the spatial resolution and the penetration depth is severely limited due to strong absorption (Warther et al., 2010). The development of two-photon responsive glutamate cages has allowed both finer spatial resolution and deeper penetration into tissue (Matsuzaki et al., 2001), as uncaging can occur with exposure to 800 nm light. Further refinement has reduced the optical radiation required by improving the efficiency of the two-photon uncaging process (Warther et al., 2010) or developing cages that release upon exposure to visible light (Verde et al., 2008; Fino et al., 2009). Caged molecules are also able to inhibit neural activity through the release of GABA (Verde et al., 2008; Warther et al., 2010). However, this technique requires further development, as many of the compounds used also interact with the receptor before photoactivation (Warther et al., 2010).

## 5 Other nanoparticle modalities

While this review has primarily focused on optically-responsive nanoparticles, these versatile materials have also been used to transduce other forms of energy for neuromodulatory effects. For example, it has been shown that magnetic-field heating of superparamagnetic ferrite nanoparticles can be used to remotely activate temperature-sensitive cation channels in cells (Huang et al., 2010). Similar to the approach of Nelidova et al. (2020) (Section 4.1), the ferrite nanoparticles were targeted to TRPV1 channels on the plasma membrane of cells but were heated by a radio-frequency magnetic field. In addition to thermal modulation, magnetic nanomaterials can be used to transduce mechanical (Tay and Di Carlo, 2017; Gregurec et al., 2020) and chemical effects (Romero et al., 2016; Rao et al., 2019) in neuronal cells. Therefore, given that magnetically-responsive nanoparticles or exogenous proteins can significantly enhance the coupling between minimally-invasive external electromagnetic devices and any neurons in close proximity to the magnetic materials, this modality should not be discounted (Christiansen et al., 2020). While the size, spatial resolution and power consumption of current magnetic coils appears unsuitable for a head-mounted retinal prosthesis, more invasive miniaturized coils have been developed to activate select groups of neurons (Bonmassar et al., 2012; Lee et al., 2016; Rizou and Prodromakis, 2018).

While ultrasound neuromodulation has been discussed in Section 3.3 and photoacoustic effects in Section 4.2, acoustic fields can also be used to induce electrical responses through the piezoelectric effect in certain nanoparticles (Cafarelli et al., 2021). This is an interesting extension of the various enhancing effects that can be achieved by combining nanoparticles with less invasive energy sources and is mentioned here for completeness. However, this field is still in its infancy and we are not aware of any applications of this approach in the retina.

## 6 Nanoparticle pharmacokinetics in the retina

Nanoparticles can be designed and tuned to navigate a variety of biological microenvironments, negotiate biological barriers, and deliver therapeutics or diagnostic agents to specific cells and tissues in the body (Anselmo and Mitragotri, 2021). Several nano- and micro-particles have been approved by the Food and Drug Administration for human use with delivery by oral, local, topical, and systemic (e.g., intravenous) administration, depending on the desired application or targeted site. Sharma et al. (2021) have reviewed various emerging nano-biomaterials, such as nanoparticles, nanowires, hybrid nanostructures, and nanoscaffolds, that have been used for ocular tissue engineering and retinal regeneration in mice. The interactions of nanomaterials with biological systems are known to depend on properties such as size, shape, chemical functionality, surface charge, and composition (Albanese et al., 2012). It has been noted that several currently approved nanotherapeutics exhibit fewer side effects than their small molecule counterparts, while other nanoparticles tend to display toxicity (Wolfram et al., 2015; Scheive et al., 2021). A complete discussion of these complex interactions is beyond the scope of this review, which instead focusses on what is known about nanoparticle delivery to the retina.

**Figure 4:**
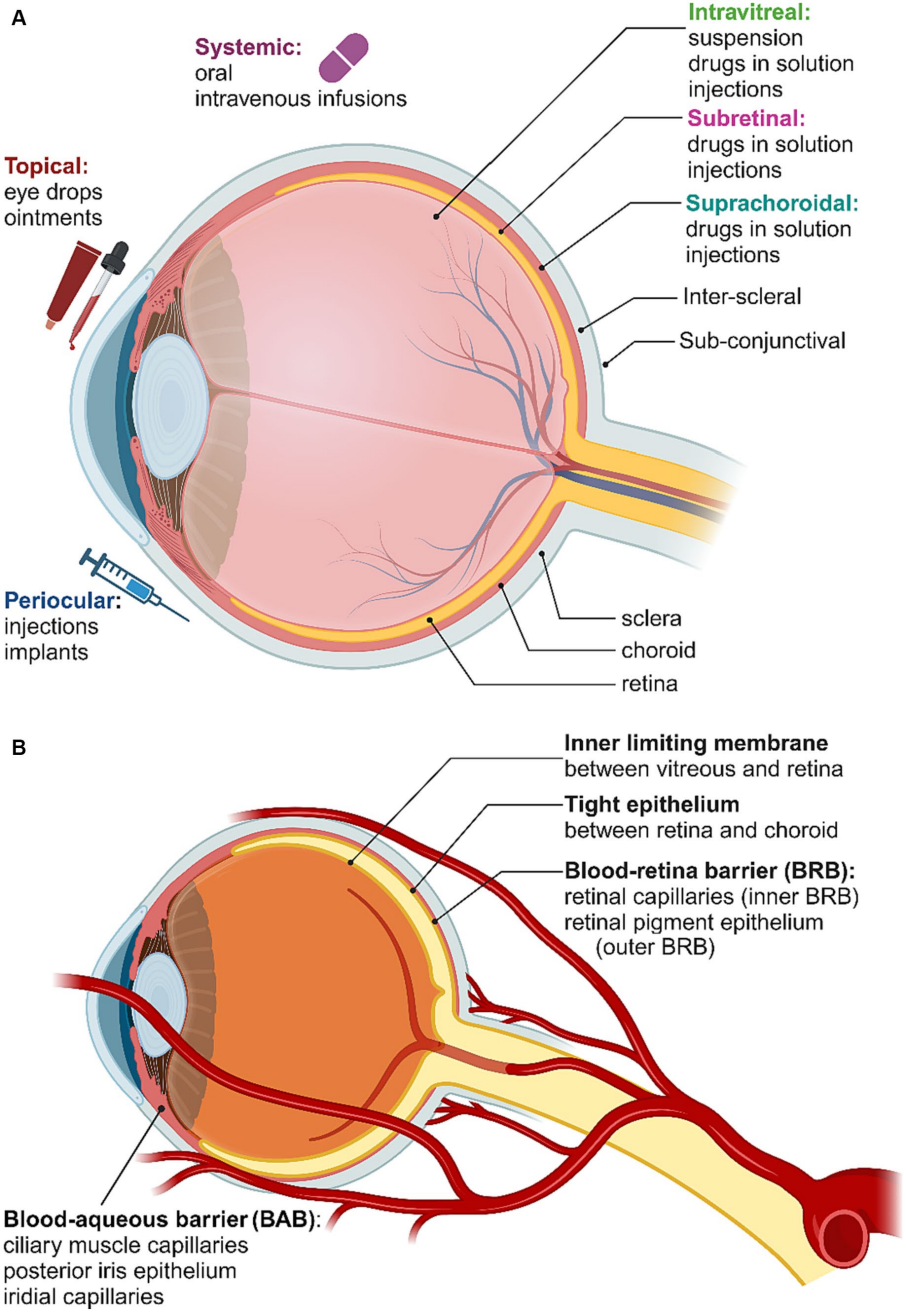
**(A)** Routes of administration for retinal drug delivery. **(B)** Blood-ocular barriers. Created in Biorender.com (2023).

A variety of obstacles and biological barriers have to be overcome to deliver drugs and other therapeutic agents to the retina. The eye is divided into two compartments: the anterior segment, which includes the cornea, iris, pupil, ciliary body and conjunctiva, and the posterior segment, which includes the sclera, choroid, fovea, vitreous humor, optic nerve and retina. Each of the possible delivery pathways to the retina are depicted in Figure 4A, while the various barriers to retinal drug delivery are summarized in Figure 4B. The known interactions between nanoparticles and these pathways and barriers will be briefly outlined here, with the reader directed to detailed reviews elsewhere (del Amo et al., 2017; Loftsson, 2022; Tawfik et al., 2022). Some key examples of nanoparticles that have been employed for drug delivery or therapeutic effects in the retina are summarized in Table 1.

Topical delivery via eye drops or ointments is the most patient-friendly path, but is relatively inefficient for several reasons, including dilution by the tear film and the long diffusion path from the cornea to the retina. Nonetheless, it has been shown that eye drops containing solid drug/cyclodextrin complex microparticles with a mean diameter of 2–4 μm and dissolved drug/cyclodextrin complex nanoparticles can deliver significant amounts of drugs to the posterior segment (Loftsson and Stefansson, 2017). There appears to be considerable scope to explore these kinds of cyclodextrin nanocarriers for self-administered delivery of other nanoparticles to the retina (Lorenzo-Veiga et al., 2021). Iontophoretic drug delivery is also considered non-invasive, using low current flow to deliver drugs through the ocular barriers. For retinal delivery, the trans-scleral pathway can be used to avoid anterior segment barriers. Although iontophoresis appears better suited to small molecule drugs rather than nanoparticles, micellar carrier systems, in which the particle is surrounded by a self-assembled monolayer of amphiphilic molecules, have also been investigated in this context (Chopra et al., 2012).

Subretinal injection targets the space between the RPE and the photoreceptors. Direct contact with the photoreceptors makes this a preferred site for drug delivery for the treatment of retinal degenerative diseases. Although this invasive route of delivery is a simpler surgical operation than the implantation of a retinal prosthesis, it remains technically demanding, particularly in low-resource settings. The risk of temporary focal retinal detachment and the creation of a retinotomy is raised, particularly for patients whose retinal cellular integrity has already been compromised (Tawfik et al., 2022). Here the outer limiting membrane, which is formed between the apical processes of the Müller cells and the inner segments of the receptors, may create a barrier to nanoparticle transport, which can be disrupted in pathological conditions (Omri et al., 2010). Despite these limitations, subretinal delivery to the photoreceptors and outer nuclear layer has been successfully demonstrated in some early-stage experiments. In the work of Nelidova et al. (2020), subretinal injection was used to deliver antibody-conjugated gold nanorods together with adeno-associated virus carrying TRPV1 transgenes modified with the 6x-His epitope tag. The gold nanorods formed stable associations with the TRPV1 channels when they expressed on the remaining photoreceptor cell bodies of blind mice and in *ex vivo* human retinas. Safety was assessed by showing that near infrared light failed to activate microglia, or reduce retinal layer thickness, opsin density, or cone density, whereas the nanorods failed to activate microglia, increase apoptosis, or reduce retinal layer thickness when assessed 100 days after injection. Similarly, semiconducting polymer nanoparticles of 300 nm diameter were widely and persistently distributed over the entire subretinal space for up to 8 months after subretinal injection in rats (Maya-Vetencourt et al., 2020). These nanoparticles showed no signs of migrating toward the inner retinal layers and were found to trigger neither trophic nor proinflammatory effects.

Intravitreal injection is the most commonly used administration route and generally provides good bioavailability. While intravitreal injection is significantly less technically demanding than subretinal delivery (Tawfik et al., 2022), it remains invasive and uncomfortable. The vitreous humor is comprised of the anionic hydrophilic polymer hyaluronan and embedded collagen fibers that provide strength and resistance to external forces. The vitreous has a relatively open structure and the diffusivity of fluorescently labeled polystyrene nanoparticles has been measured in the bovine vitreous using particle tracking techniques. The diffusion coefficient of neutral PEG-coated nanoparticles of size up to 500 nm was found to be about two times smaller than in water, whereas the diffusivity of anionic nanoparticles up to 200 nm was approximately two times higher than in water (Xu et al., 2013).

In contrast to diffusion, convective flow towards the retina is negligible compared to the aqueous humor flow in the anterior chamber and does not appear to play an important role in nanoparticle transport. The velocity of posterior fluid flow across the retina has been estimated to be less than 2 × 10^−5^ cm/min in rabbit eyes (Araie and Maurice, 1991). The RPE forms part of the blood retina barrier and is expected to block transport from the vitreous cavity to the choroid. Nonetheless, osmotic pumping of the RPE, which prevents detachment of the retina from the choroid, may play some role in transport of nanoparticles across the retina. While the water dynamics in the eye, particularly the posterior segment, remain relatively poorly understood, it has been shown that the water channel aquaporin-4, which is abundant in the Müller cells of the retina, plays a role in regulating water flow through the vitreous body (Ueki et al., 2021).

The 10–20 μm thick inner limiting membrane (ILM) presents the main barrier for delivery from the vitreous to the retina. The ILM is located between the end feet of the Müller cells and the vitreous. It is mainly composed of collagen and anionic glycosaminoglycans, which provide a mechanical and electrostatic barrier. Pore size of the ILM has been estimated to be approximately 6 nm, which corresponds to a molecular weight of about 75 kDa based on trans-retinal diffusion of FITC-dextrans (Jackson et al., 2003). However, the efficacy of NP penetration through the vitreous and ILM varies for different nanoparticles. Human serum albumin nanoparticles were injected in anionic or cationic forms to determine the effect of surface charge on intravitreal nanoparticle movement. It was found that anionic particles (zeta potential = −33.3 ± 6.1 mV) diffused more easily through the 3-dimensional vitreal network of collagen fibrils than the cationic particles (11.7 ± 7.2 mV) and were preferentially taken up into Müller cells (Kim H. et al., 2009). Another study investigated the movement of various polymeric nanoparticles with similar size (230–345 nm) but different surface charge (ranging from +33 to −26 mV). The most cationic particles interacted strongly with the anionic collagen fibrils of the vitreous, whereas two anionic particle types were able to penetrate all the way across the retina to the RPE. Cationic particles with anti-fouling glycol coatings were able to penetrate the vitreal barrier and reach the inner limiting membrane, but they did not pass through the physical pores of the ILM into the retinal structure (Koo et al., 2012). Gan et al. (2013) reported that hyaluronan-modified core-shell liponanoparticles with higher hyaluronan grafting density (5.8%) and higher molecular weight (200–400 kDa, 320 nm, −25 mV) were more effectively taken up in the RPE, while chitosan nanoparticles (190 nm, +36 mV) were limited to the vitreous cavity and bare core–shell liponanoparticles (190 nm, −10 mV) only reached the inner layers of the retina.

Fluorescent magnetic nanoparticles with negative surface charge and a hydrodynamic size of 252 nm were intravitreally injected in Xenopus embryos and zebrafish, showing high retention in the injected eye and low toxicity (Giannaccini et al., 2014). In these studies, migration of the nanoparticles from the vitreous to the RPE was complete after 24 h. In another study, cerium oxide nanoparticles (3–5 nm) were sustained in the murine retina for more than 1 year after administration without signs of inflammation or other side effects (Chen et al., 2006; Kyosseva and McGinnis, 2015). As discussed in Section 4.1, optoporation has been used to deliver fluorescently tagged siRNAs or fluorescein isothiocyanate-dextran dye into retinal cells via intravitreally-injected gold nanoparticles (Wilson et al., 2018). A femtosecond laser with 800 nm, 100 fs pulses was used to locally optoporate RGCs targeted by gold nanoparticles functionalized with an antibody toward the cell-surface voltage-gated K^+^ channel subunit K_V_1.1. Neither the gold nanoparticles nor the laser irradiation resulted in RGC death. Reflected light microscopy was used to confirm that the antibody-functionalized 100 nm gold spheres had penetrated the ILM.

Suprachoroidal injections are made under the conjunctival membrane that lines the inner surface of the eyelid. This provides a less invasive local delivery route that requires no anesthesia and avoids the cornea and conjunctiva. Delivery of nanoparticles to the retina via the suprachoroidal space is likely to be limited by rapid clearance to the high blood flow in the choroid (43 mL/h in humans) and leakiness of the choriocapillaris (70–80 nm holes) (del Amo and Urtti, 2015), versus the low permeability across the RPE. Indeed, there is some evidence that polystyrene particles 20 nm to 10 μm in size are not effectively cleared from the suprachoroidal space after 1–2 months (Patel et al., 2012). Retinal availability of nanoparticles is expected to be further reduced for the various periocular delivery routes, which include subconjunctival, peribulbar, retrobulbar and sub-tenon injections (del Amo et al., 2017). Therefore these approaches will not be discussed further here.

Systemic delivery can be achieved via oral or intravenous delivery routes. Systemic administration is seldom used for retinal delivery due to the blood-retina barrier and the significant dilution effect of the vitreous humor (volume 4–5 mL) versus the entire blood volume (approximately 5 L). However, nanotechnology-based drug delivery systems have been proposed as a means to overcome ocular physiological barriers based on transitory blood-ocular breakdown (Occhiutto et al., 2012). Kim J. H. et al. (2009) have demonstrated that intravenously administered 20 nm gold nanoparticles were able to pass through the blood-retinal barrier and were distributed into all retinal layers (75 ± 5% in neurons, 17 ± 6% in endothelial cells and 8 ± 3% in peri-endothelial glial cells). In contrast, 100 nm nanoparticles were not detected in the retina. The absence of toxicity was noted in retinal endothelial cells, astrocytes and retinoblastoma cells (Kim J. H. et al., 2009). Liposomes, which are microscopic lipid vesicles designed to encapsulate drugs, can be targeted by light delivery to the retina after intravenous injection. Absorption of light by blood and melanin in a rat model of AMD served to warm up the RPE, choriocapillaris and choroidal neovascularization to 41°C, whereupon the liposomes underwent a phase change and released their contents (Zeimer and Goldberg, 2001). Eom and Park are exploring the use of focused ultrasound with microbubbles to sonoporate retinal capillaries for delivering metallic nanoparticles of various sizes and shapes to RGCs (Eom, 2023).

As we have seen, nanoparticles can be functionalized with a variety of ligands such as small molecules, surfactants, dendrimers, polymers, and biomolecules. Surface functionalization can be used to control cellular internalization, cytotoxicity, binding capacity and immunogenicity (Mout et al., 2012). A study of an *in vitro* tissue culture model of the mouse retina exposed to low concentrations of citrate-stabilized 20 and 80 nm gold nanoparticles found cellular and nuclear uptake of NPs in all neuronal layers of the retina, morphological disruption of the normal complex layered retinal structure, vacuole formation and pyknotic cells after exposure, significantly higher numbers of apoptotic cells, an increased number of oxidative stressed cells and increased microglial cell activation (Söderstjerna et al., 2014). However, it is known that cell viability is reduced with exposure to higher concentrations of citrate on the particle surface (Freese et al., 2012) and 155-nm diameter gold nanoshells coated with polyethylene glycol have shown no indication of toxicity in a wide range of *in vitro* and *in vivo* studies (Gad et al., 2012). A mechanistic understanding of the cytotoxicity of surface modified gold NPs is starting to emerge, with the suggestion that toxic gold NPs share a common structural characteristic of a hydrophobic moiety neighboring a positive charge (Lee et al., 2019).

Cell-specific targeting with antibody-coupled nanoparticles requires a cell-specific antigen on the cell surface. However, the networked structure of the retina raises further complications. For example, as discussed in Section 2, the axons of RGCs project radially from the cell body across the retinal surface to converge at the optic disk. If antibody-conjugated nanoparticles bind to both cell somas and axons, it may become difficult to modulate a specific cell without influencing RGC cell bodies located distally in the retina via their axonal projections. In the absence of any known surface-expressed soma-specific antigens on RGCs, Wilson et al. (2018) addressed this issue by targeting nanoparticles to K_V_1.1 channels, which are predominantly expressed by RGC somas. Polyampholyte-coated CdSe/ZnS core/shell quantum dots (Walters et al., 2012) and organically modified silica (ORMOSIL) (Barandeh et al., 2012) have been shown to preferentially target neurons. A wider range of cell- and location-specific targets is required to support accurate, effective and stable delivery of nanoparticles to the retina.

## 7 Computational modeling of nanoparticle-based optical interfaces

Computational modeling of neurons can be an invaluable tool in guiding electrophysiology experiments and reducing the number of required animals (Dayan, 2005). Computer simulations can uncover underlying mechanisms observed during a particular biological phenomenon, make solid predictions on how a neuron might respond to stimuli, and can also help to constrain the stimulation parameter space, which is more difficult to achieve experimentally.

Computational models are necessarily a simplification of a complex cell biology, and the level of detail one chooses to simulate is dependent on the observed electrophysiological phenomenon. Complex multi-neuronal networks, for example, are essential for reproducing behavior arising from interactions between neurons, and a wide range of feedback and inhibitory models exist to replicate such behaviors in the retina (Smith and Vardi, 1995; Hosoya et al., 2005; Wohrer and Kornprobst, 2009; Martínez-Cañada et al., 2015). For the study of individual neurons, a range of approaches may be used of various complexity, from simple integrate-and-fire (Burkitt, 2006) and Izhikevich models (Izhikevich, 2003), to more complex Hodgkin-Huxley representations (Hodgkin and Huxley, 1952).

Despite substantial diversity in the types of neurons which comprise the retina, the highly-organized nature of its circuitry constitutes complex but theoretically-tractable electrophysiological architecture, which has been studied extensively for the purpose of elucidating the underlying mechanisms of a wide range of single-cell and network response dynamics (Guo et al., 2014). Substantial bodies of literature exist for computational modeling of all major retinal neuron types, including photoreceptors (Kamiyama et al., 1996; Publio et al., 2006), bipolar cells (Usui et al., 1996), amacrine cells (Tarchick et al., 2023) and RGCs (Fohlmeister and Miller, 1997; Kameneva et al., 2011). For a review of computational models of the retina, we refer the reader to (Guo et al., 2014).

None of the retina models mentioned above consider the thermal properties of nanoparticle-based optical stimulation. The primary electrophysiological mechanisms underpinning nanoparticle-enhanced infrared neural modulation in particular are thermal, not optical, in nature. Eom et al. (2018) proposed an integrated model that related the heat generated by near infrared light interacting with gold nanorods GNRs to the resulting effects on temperature-dependent TRPV1 ion channels and a heat-induced capacitive current across the membrane. The laser-evoked heating was calculated first for a single nanorod, and then extrapolated to a uniform monolayer distribution on the tissue surface.

Mathematical modeling can also be used to predict the pharmacokinetics of nanoparticles in biological settings. For a recent review of computer simulations of nanoparticles interacting with a cell membrane, we refer the reader to Zhang X. et al. (2021). The approaches for modeling nanoparticles are also covered in detail in one of the chapters in the book focused on computer aided pharmaceutics and drug delivery (Bicak et al., 2022). Similarly, physiologically-based pharmacokinetic modeling of nanoparticles is covered in Yuan et al. (2019). The latest multi-scale simulations of nanoparticle transport barriers, with a focus on cancer medicine, are reviewed in Stillman et al. (2020).

## 8 Conclusion

The nanoparticle-based optical interfaces for retinal neuromodulation reviewed here show promise for the development of prosthetic vision with greatly improved visual acuity and reduced invasiveness. Implicit in this approach is that key components such as head-mounted cameras, light projection goggles, signal processors and power packs can be worn externally, where they can more readily be serviced and upgraded as technology advances. Selective neuromodulation at a cellular level will almost certainly require high accuracy eye tracking (Paraskevoudi and Pezaris, 2019). Although the performance and size of eye tracking technologies is likely to continue to improve, alternative approaches based on intraocular cameras positioned in place of the crystalline lens have also been proposed (Stiles et al., 2010).

The detailed requirements for light projection and image processing to appropriately encode information at different levels of the extant retinal circuitry for correct interpretation in the visual cortex are currently not well understood. Similarly, the optimal performance specifications of a nanoparticle-based retinal interface – in terms of spatial and temporal resolution, response dynamics and exposure to long and repeated stimulation – cannot be confidently specified at this stage. While the recent results reported for photothermal stimulation and inhibition of RGCs by gold nanoparticles (Begeng et al., 2023) suggest that a high degree of spatial and temporal control is theoretically possible, there will almost certainly be tradeoffs, for example in terms of heat buildup during repeated stimulation. Moreover, further investigation is required to establish whether RGCs become desensitized to repetitive stimulation via nanoparticle interfaces, as has been observed for electrical stimulation with conventional retinal prostheses (Jensen and Rizzo, 2007; Freeman and Fried, 2011; Soto-Breceda et al., 2018). Deployment of the nanoparticles themselves requires further work to ensure delivery of nanoparticle tools at appropriate levels to the most appropriate cell types and cellular sites. As discussed in Section 3.1, these kinds of technologies, parameters and constraints are currently under investigation for optogenetic approaches, which are relatively further advanced towards clinical translation. Nanoparticle-based approaches therefore appear likely to benefit from the adjacent technologies developed for optogenetics. As our understanding of the opportunities and limitations of the different technologies progresses, hybrid approaches that “mix and match” genetic and nanoparticle-based tools might also emerge.

In order to translate optical approaches into retinal prostheses, a greater understanding of both the mechanisms of optical modulation and the engineering limitations is desirable. For example, with standard electrophysiology approaches, it is not possible to directly compare the cell’s response to a test pulse after an optical or electrical stimulation that leads to the same depolarization. It has been proposed that a dynamic optical clamp is needed to enhance control and to facilitate future investigations of ion channel dynamics during optical stimulation (Hart et al., 2023). This approach might also support the development of closed-loop neuronal control in future generations of optical neuroprosthetic devices. The “feedback” for the closed loop system could potentially be provided by mapping the visual field via visually-evoked responses in real time with a non-invasive cortical imaging technique such as multifocal magnetoencephalography (Nishiyama et al., 2004; Crewther et al., 2016).

The discussion in Section 6 confirmed that the safety and bioavailability of nanoparticles is highly dependent on the size, shape, chemical functionality, surface charge, and composition of the particles. The complexity can be further illustrated by the conflicting evidence for the safety of quantum dot nanoparticles, which have attracted significant attention for their optical and electronic properties. Despite research suggesting no adverse effects 90 days after intravenous administration of QDs in primates (Ye et al., 2012), there is also evidence of QD quenching, chemical degradation and heavy metal leakage in the presence of cell metabolites (Mancini et al., 2008). In general, it appears that further investigations into biodegradability, clearance, and toxicity will be required for each specific formulation of nanoparticle, and that the critical quality attributes of any approved nanotherapeutic will have to be defined and tightly controlled for reproducible manufacturing at scale (Đorđević et al., 2022).

Phototoxic effects, defined as effects on the retina related to light incident on the retina, can be distinguished in terms of photothermal, photomechanical and photochemical toxicity effects. These have been reviewed in detail elsewhere (Lawwill et al., 1977; Youssef et al., 2011; Hunter et al., 2012). In brief, the potential of a certain light stimulus to induce phototoxic damage mainly depends on the energy delivered, which is a function of its intensity and wavelength. Consensus agreement has been achieved regarding acceptable levels of light exposure to the eye [International Commission on Non-Ionizing Radiation Protection (ICNIRP), 2013] and these guidelines have been widely incorporated into national legislations. Any nanoparticle-based optical interface for the retina that requires stimulation by light intensifying projectors would need to operate within these established ocular exposure safety thresholds.

An important challenge that has emerged from recent work is the need to measure temperature at nanoscale and tissue macroscale in order to understand the damage risk profile in more detail, and to clearly differentiate between photothermal and other effects. This is especially important because all the non-thermal nanoparticle-mediated transduction processes necessarily rely on optical absorption, so any energy that is not directly converted into acoustic, electrical or chemical energy is likely to generate heat as a byproduct.

Ultimately, the full potential of these emerging nanotechnologies for retinal neuromodulation may only become clear once we have a more detailed understanding of the neural code of vision and visual plasticity (Abbasi and Rizzo, 2021), including the remodeling that occurs in the diseased retina at all stages of degeneration. In their investigation of the retinal remodeling process in humans with retinitis pigmentosa, Jones et al. (2016) observe that their “results suggest interventions that presume substantial preservation of the neural retina will likely fail in late stages of the disease. Even early intervention offers no guarantee that the interventions will be immune to progressive remodeling. Fundamental work in the biology and mechanisms of disease progression are needed to support vision rescue strategies.” While these are important caveats, they also support the use of less invasive modalities that could in principle be applied in a more flexible and adaptable fashion as the disease progresses.

## Author contributions

PS: Writing – original draft, Conceptualization. JB: Writing – original draft; WT: Writing – review & editing; MI: Writing – review & editing; TK: Writing – original draft, Visualization.
